# Detection and Magnetic Source Imaging of Fast Oscillations (40–160 Hz) Recorded with Magnetoencephalography in Focal Epilepsy Patients

**DOI:** 10.1007/s10548-016-0471-9

**Published:** 2016-01-30

**Authors:** Nicolás von Ellenrieder, Giovanni Pellegrino, Tanguy Hedrich, Jean Gotman, Jean-Marc Lina, Christophe Grova, Eliane Kobayashi

**Affiliations:** Department of Neurology and Neurosurgery, Montreal Neurological Institute and Hospital, McGill University, 3801 University Street, Montreal, QC H3A 2B4 Canada; Multimodal Functional Imaging Lab, Biomedical Engineering Department, McGill University, 3775 University Street, Montreal, QC H3A 2B4 Canada; LEICI, CONICET – Universidad Nacional de La Plata, Calle 116 y 48, 1900 La Plata, Argentina; Département de Génie Électrique, École de Technologie Supérieure, 1100 Notre-Dame Street West, Montreal, QC H3C 1K3 Canada; Centre de Recherches Mathematiques, Univeristé de Montréal, 2920 Chemin de la tour, Montreal, QC H3T 1J4 Canada; Center for Advanced Research on Sleep Medecine, Centre de Rech. de l’Hôpital du Sacré-Cœur de Montréal, 5400 W Gouin Blvd, Montreal, QC H4J 1J5 Canada; Physics Department and PERFORM Centre, Concordia University, 7141 Sherbrooke Street West, Montreal, QC H4B 1R6 Canada

**Keywords:** Epilepsy, Human, High frequency activity, Automatic detection, Source localization

## Abstract

We present a framework to detect fast oscillations (FOs) in magnetoencephalography (MEG) and to perform magnetic source imaging (MSI) to determine the location and extent of their generators in the cortex. FOs can be of physiologic origin associated to sensory processing and memory consolidation. In epilepsy, FOs are of pathologic origin and biomarkers of the epileptogenic zone. Seventeen patients with focal epilepsy previously confirmed with identified FOs in scalp electroencephalography (EEG) were evaluated. To handle data deriving from large number of sensors (275 axial gradiometers) we used an automatic detector with high sensitivity. False positives were discarded by two human experts. MSI of the FOs was performed with the wavelet based maximum entropy on the mean method. We found FOs in 11/17 patients, in only one patient the channel with highest FO rate was not concordant with the epileptogenic region and might correspond to physiologic oscillations. MEG FOs rates were very low: 0.02–4.55 per minute. Compared to scalp EEG, detection sensitivity was lower, but the specificity higher in MEG. MSI of FOs showed concordance or partial concordance with proven generators of seizures and epileptiform activity in 10/11 patients. We have validated the proposed framework for the non-invasive study of FOs with MEG. The excellent overall concordance with other clinical gold standard evaluation tools indicates that MEG FOs can provide relevant information to guide implantation for intracranial EEG pre-surgical evaluation and for surgical treatment, and demonstrates the important added value of choosing appropriate FOs detection and source localization methods.

**Electronic supplementary material:**

The online version of this article (doi:10.1007/s10548-016-0471-9) contains supplementary material, which is available to authorized users.

## Introduction

High frequency oscillations (HFOs) are biomarkers of the epileptogenic zone in intracranial electroencephalography (iEEG) recordings of focal epilepsy patients (Bragin et al. 2010; Gotman 2010; Worrell and Gotman 2011; Zijlmans et al. 2012; Jacobs et al. 2012). To expand the clinical utility of these biomarkers to a broader patient population it is of interest to study them with non-invasive techniques such as scalp EEG or magnetoencephalography (MEG). The use of these non-invasive techniques could also shed light into the unknown differentiation of physiologic versus pathologic HFOs by allowing a comparison to healthy controls, an approach not available for invasive methods traditionally employed in the study of high frequency oscillations.

Despite the attenuation and blurring effect of the skull and the potential susceptibility to artifacts such as muscle activity, studies in scalp EEG consistently found oscillations in the high-gamma (40–80 Hz) and ripple (80–200 Hz) bands, with lower rates than in intracranial EEG recordings (Kobayashi et al. 2004; Andrade-Valença et al. 2011; Melani et al. 2013; Zelmann et al. 2014; Fahoum et al. 2014). To distinguish events with frequencies typically studied with intracranial EEG (80–500 Hz) from events that can be recorded in scalp EEG with frequencies starting in the gamma band, we will refer to the former as HFOs and to the latter as fast oscillations (FOs). Note that FOs of pathologic origin in the gamma band can also be invasively recorded (Rampp et al. 2010; Ren et al. 2015).

There are few studies of epilepsy patients evaluating MEG high frequency activity. These include investigation of the power in high frequency bands of patients with generalized epilepsy (Miao et al. 2014; Tenney et al. 2014), as well as the high frequency content at the time of spikes (Guggisberg et al. 2008; Rampp et al. 2010). Regarding short oscillations standing out of the background, one study investigated FOs in the gamma band in patients with focal cortical dysplasia and reports rates of around two per minute (Jeong et al. 2013). Another recent study investigated FOs in the ripple band (van Klink et al. 2015), they found ripples in three out of 12 patients, and showed an increase of sensitivity to FOs by using virtual channels constructed using beamforming techniques based on information obtained from spikes.

In this study we present a practical approach for the detection of FOs in the high-gamma (40–80 Hz) and ripple (80–160 Hz) band in MEG signals derived from 275 axial gradiometers, completely independent of the occurrence of interictal epileptic discharges (IEDs). We test this approach in epilepsy patients who underwent a previous scalp EEG FO study, allowing us to compare feasibility and performance in both modalities (Andrade-Valença et al. 2011; Melani et al. 2013).

In EEG recordings it is a common approach to detect HFOs visually. This is feasible and practical in iEEG data given the high HFO rates in this type of signals (at times greater than 60 per minute), which allow to analyze only a few minutes of recordings and still get consistent results (Zelmann et al. 2009). In scalp EEG the lower rates (highest observed rates between 5 and 10 per minute, Andrade-Valença et al. 2011) require analysis of longer recording times. Analysis of this type have been done only in montages with a low number of sensors, so visual detection of FOs is still practical. In standard MEG recordings a purely visual detection of the events is unpractical if not impossible because the expected FO rates are lower than in iEEG, and the number of sensors is higher than in scalp EEG (higher than 100 and up to 275 in whole head dewars). The approach we adopt is to use an automatic pre-detection step with high sensitivity, followed by a visual review of the candidate events to discard false positives.

The use of source localization techniques to study FOs non-invasively is of great importance, in epilepsy patients to determine the focus, and in healthy subjects to study physiologic FOs, an approach not available for invasive methods traditionally employed in the study of high frequency oscillations. The low number of electrodes used in scalp EEG studies so far complicates the use of source localization techniques in this modality. On the other hand, in MEG the large number of sensors represents an advantage in this aspect. Magnetic source imaging (MSI) of MEG FOs is nevertheless a challenging task, since only a few events with poor signal to noise ratio (SNR) are expected to be available. MSI of the gamma band content of spikes has been performed using beamforming techniques (Guggisberg et al. 2008), and source of FOs in the gamma band were localized using sLORETA (Jeong et al. 2013). To our knowledge MSI of FOs in the ripple band has not been reported. In this study we used the Maximum Entropy on the Mean (MEM) method to localize the sources of the detected FOs in the high-gamma and ripple band. The MEM is an efficient technique that has been successfully used for determine the location and extent of sources of epileptic activity (Chowdhury et al. 2013; Heers et al. 2014, 2015). For MSI of FOs we employ the wavelet MEM (wMEM) method, an extension of MEM particularly well suited for this application since it was developed precisely for localizing oscillatory activity (Lina et al. 2014).

## Methods

### Patient Selection

In this retrospective study we included patients who (i) had a previous positive investigation of scalp EEG FOs (Andrade-Valença et al. 2011; Melani et al. 2013), and (ii) underwent a separate simultaneous EEG-MEG 1-h recording session. Seventeen patients fulfilled the inclusion criteria. The assessment of scalp EEG FOs in nine of them (Patients 1–5 and 10–13) was in the context of a project in which the inclusion criterion was focal epilepsy with at least one IED per minute during a preliminary scalp EEG study (Andrade-Valença et al. 2011). The remaining eight patients belonged to a project in which the only inclusion criterion was focal epilepsy (Melani et al. 2013). Demographics and clinical information are given in Supplementary Table 1. This study was approved by the Montreal Neurological Institute Research Ethics Board and all patients signed a written informed consent prior to the study.

### Epileptogenic Region

To define a gold standard for the source localization results, two specialists defined the epileptogenic region based on the available clinical information for each patient. This information is included in Supplementary Table 1, and consisted of (in order of priority; not all factors were available for every patient): resected region, ictal and interictal iEEG findings, visible lesion in the MRI, ictal and interictal scalp EEG findings. The epileptogenic region for each patient was independently marked by both reviewers, and a consensus was reached by the experts after discussing the cases in which there were differences (all differences were restricted to the extent of the epileptogenic region, with no patients showing discordant regions). The reviewers were blind to the source localization results at the moment of marking the epileptogenic zone. The marking was done over the inflated brain derived from each patient’s MRI, visualized in 6 orientations.

### MEG Data Acquisition and Pre-processing

Simultaneous EEG/MEG recordings were performed at the Montreal Neurological Institute, McGill University and at the Department of Psychology, Université de Montreal using a 275 sensor CTF-MEG-system (MISL, Vancouver, Canada) and a 56 sensor EEG-cap with ceramic electrodes (Easy-cap, Herrsching, Germany). The sampling rate was 1200 Hz in one patient and 2400 Hz in the remaining patients. The duration of the recordings was constrained by each patient’s ability to stay still in the dewar. The data was acquired in blocks of 6 min duration, with a median of 9 blocks per patient (range 6–11), i.e. median total recording time of 54 min (range 36–66 min). Since this project aimed to characterize interictal FOs, we excluded the blocks containing ictal activity from two patients who had seizures during the MEG acquisition. All the recordings were downsampled to 640 Hz. The technical details of the data processing can be found in Appendix.

### Automatic Detection

The MEG automatic FOs detector was based on an algorithm developed for scalp EEG (von Ellenrieder et al. 2012). It searched for an increase in the root mean square (RMS) amplitude of the signal in narrow frequency bands, and compared it to the background in a 5 s sliding window. A detection occurred when the RMS amplitude in any narrow frequency band was at least 2.5 times larger than the background during an interval longer than 4 cycles of the central frequency of the band plus the effective duration of the narrowband filters impulse response. There were ten narrow bands in the 40–160 Hz range (40–46–53–61–70–80–93–107–123–142–160 Hz), five in the high-gamma band and five in the ripple band. The filter details are given in Appendix. The automatic detector was set to work with high sensitivity in order to avoid missing any true FO. Since the high sensitivity implies low specificity, a high proportion of false positives was expected at this step. We will refer to the candidate FOs found in this first step as ***pre*-*detections***. To exclude muscle activity and movement artefacts, all pre-detections that fell in one of the following three categories were discarded: (i) Pre-detections that occurred simultaneously in more than 200 narrow frequency bands and channels, or in more than 100 channels. This criterion relies on the fact that muscle activity and movement artefacts usually involve many channels and have broad frequency content (Jeong et al. 2013). (ii) If the channel with highest rate of pre-detections was located at the edge of the helmet all pre-detections in this channel were excluded, as well as all pre-detections occurring at the same time in other channels. This criterion is based on empirical observations showing that the effect of movement artefacts is more pronounced in channels at the border of the helmet (Muthukumaraswamy 2013). (iii) Pre-detections in which the power increase with respect to the background in the high-gamma or ripple band was lower than in channels with no pre-detections. This led to the exclusion of potentially true positive FO events occurring simultaneously with other high frequency physiologic activity, instrumental noise, or artefacts that would have affected the source localization results. These events were excluded only to facilitate the source localization procedure.

The next step was to identify the channel with maximum rate of pre-detections for each band separately. The final output of the automatic detector consisted only of the pre-detections that occurred in any channel at the same time as a pre-detection in the highest rate channel. This approach simplifies the rest of the analysis since the visual review that follows is done only at the time of pre-detections in the highest rate channel. This is an easily replicable methodology that precludes a biased selection of a few pre-detections that might coincide with clinical information. At the same time, in epilepsy patients looking only at the channel with highest rate minimizes the detection of physiologic FOs, since the rate of pathologic FOs is higher (Matsumoto et al. 2013; Wang et al. 2013).

### Review by Experts

As mentioned above, a large number of false positive pre-detections was expected and required a second step carried out by human reviewers to visually identify and select true positive events. This task was performed on a number of pre-detections limited to 100 in the high-gamma and 100 in the ripple bands, i.e. for patients with more than 100 pre-detections in a given band, we only reviewed a random subset of 100, assuming that the sample was representative of all the detected events.

The visualization of the pre-detections for review was done using Brainstorm (Tadel et al. 2011), which is documented and freely available for download under the GNU general public license (http://neuroimage.usc.edu/brainstorm). The reviewers were presented with with 1-s epochs centered around the pre-detections, showing the bandpass filtered signal in the high-gamma (40–80 Hz) or ripple (80–160 Hz) band from the highest rate channel and 17 neighboring channels. A topographic map of the time–frequency decomposition of the signal in the high-gamma or ripple band at all channels was also presented to help identify potential muscle artifacts.

The pre-detections were independently classified by two expert reviewers (EK and GP) following the definition of HFOs commonly used in EEG investigations, i.e. four oscillations clearly standing out of the background in the analyzed high-gamma (40–80 Hz), and ripple (80–160 Hz) frequency bands (Jacobs et al. 2012, 2012; Andrade-Valença et al. 2011; Melani et al. 2013). To maximize the reliability of the detections, we admitted as true positives only the pre-detections that both reviewers considered FOs based on these criteria. The signal from FOs labelled as true positive by both experts was then carefully reviewed by one of the experts in the clinical frequency range (0.3–70 Hz) to remove any remaining detections associated to artefacts. This last step also allowed us to determine the co-occurrence of FOs and IEDs. Concordance at channel level was defined for FOs when the channel with highest FO rate was in the affected lobe.

### MSI of High-Gamma and Ripple FOs

The anatomical MRI of each patient used for MSI consisted of a T1 W MPRAGE 1 mm isotropic 3D acquisition (192 sagittal slices, 256 × 256 matrix, TE = 2.98 ms, TR = 2.3 s, Siemens Tim Trio 3T scanner). The MRI was segmented and the cortical surface obtained using BrainVISA v.4.4.0 (IFR49, France) (Mangin et al. 2004) and the MEG forward problem was solved with the boundary element method for a single layer model using OpenMEEG (Gramfort et al. 2010). Source localization of the FOs was performed with the wavelet version of the Maximum Entropy on the Mean (wMEM) method (Lina et al. 2014). We used implementations of OpenMEEG and wMEM available in the current version of Brainstorm.

The MEM principle (Amblard et al. 2004) is a non-linear inverse problem solver, coupled with a data-driven parcellation (DDP) to cluster the whole cortical surface into K non-overlapping parcels, as originally proposed by Lapalme et al. (2006). DDP consists in using partial information from the available data in order to guide this spatial clustering. The key aspect of DDP lies in the pre-localization of the sources of brain activity using the multivariate source pre-localization (MSP) method (Mattout et al. 2005). MSP is a projection method that estimates a coefficient, which characterizes the possible contribution of each dipolar source to the data. DDP in K parcels is then obtained using a region-growing algorithm around the local maxima of the MSP map. In the MEM reference model, a hidden variable is associated to each parcel in order to model the probability of the parcel to be active (probability initialized using the MSP coefficients). Parcels that do not contribute to explain the measured data are automatically switched off by maximizing the entropy of a mixed probability distribution, allowing the method to recover accurately the source location together with their spatial extent along the cortex (Chowdhury et al. 2013). This property of the MEM framework is particularly important in the context of epilepsy where spatially extended generators are expected, and we have carefully demonstrated the ability of MEM to perform accurate source reconstruction of interictal spikes in EEG and MEG (Heers et al. 2014; 2015). The wMEM extension of the MEM framework (Lina et al. 2014) decomposes the signal in a discrete wavelet basis before performing MEM source localization on each time–frequency box. Thus, wMEM is particularly suited to localize oscillatory patterns, as evaluated with realistic simulations (Lina et al. 2014).

In this study, we localized only the time–frequency box with highest amplitude during the detected FO, in the high-gamma and ripple bands separately. The resampling of the signals to 640 Hz ensured that the third scale of the discrete wavelet transform corresponded to the high-gamma band (40–80 Hz), and the second scale to the ripple band (80–160 Hz). A diagonal model was adopted for the noise covariance matrix in the data space, estimated independently for each FO. The estimation was done based on the MEG background in the high-gamma or ripple band in a 0.5 s window immediately before the beginning of each FO.

Source localization was performed for each FO. The resulting map consisted of a cortical activation value associated to each vertex of the cortical tessellation. Each map was normalized in order to get a maximum activation value equal to one for all FOs, and then the average of the activation value at each vertex was computed across all FOs. In this way a single cortical source localization map per patient and per frequency band was obtained.

For displaying the resulting maps over the cortical surface, a threshold of 30 % of the maximum activation was applied, as in previous studies (Heers et al. 2014, 2015). The concordance with the epileptogenic region was evaluated at sublobar level as concordant, partially concordant, or discordant. The partially concordant category corresponds to cases in which local maxima of the source map were found both inside and outside the clinically defined epileptogenic zone.

## Results

### Automatic Detection

**Fig. 1:**
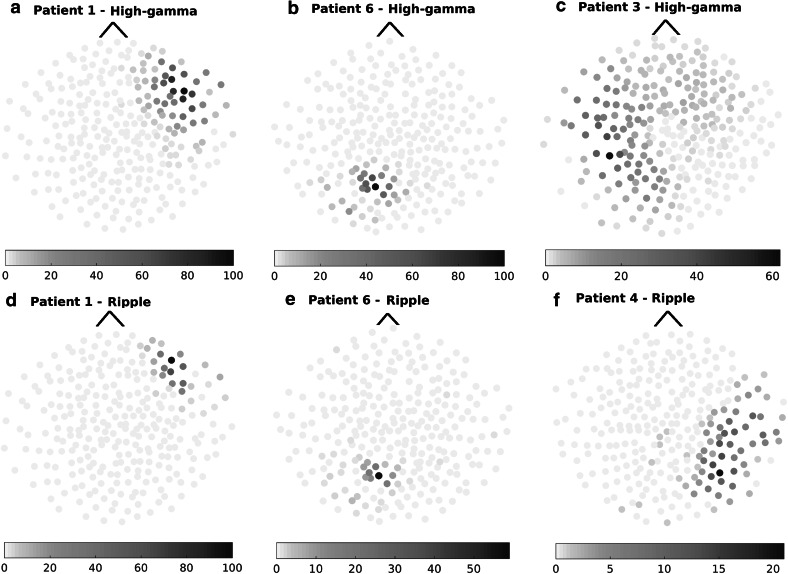
Topographic maps of the output of the automatic detector in four patients with focal epilepsy. Each MEG sensor is represented by an individual dot, the color represents number of pre-detections for each individual channel at the same time as pre-detections in the highest rate channel. *Top row* (**a**–**c**): pre-detections in the high-gamma band. *Bottom row* (**d**–**f**): pre-detections in the ripple band. **a** and **d** refer to patient 1 (right frontal epilepsy, 17 of the pre-detections were true positives in the high-gamma band and 19 in the ripple band), **b** and **e** to patient 6 (left occipital epilepsy, 5 true positives in the high-gamma band and 8 in the ripple band), **c** to patient 3 (left temporal epilepsy, 3 true positives), and **f** to patient 4 (right temporo-parietal epilepsy, 1 true positives)

The automatic detection step identified pre-detections in all 17 patients: the median number of pre-detections per patient was 32 (range 5–482) in the high-gamma band, and 21 (range 9–384) in the ripple band. The values for each patient can be found in Table 1. Figure 1 shows examples of topographic maps of the number of pre-detections in each MEG channel that occurred at the same time as a pre-detection in the channel with maximum rate. Panels E and L in Figs. S1–S17 in the supplementary material show these results for each of our patients.

**Table 1:** Characteristics of MEG fast oscillations: number of pre-detections, true positive FOs, FOs co-occurring with IEDs and concordance with epileptogenic region at channel level (highest rate channel compared to affected lobe) and source levels (source map activations compared to epileptogenic region) for high-gamma and ripple band FOs

Patient	Time (min)	MEG high-gamma oscillations (40–80 Hz)					MEG Ripple oscillations (80–160 Hz)					
		Pre-detections	FOs	FOs +IED	Concordance		Pre-detections	FOs	FOs +IED	Concordance		
					Channel	Source				Channel		Source
1	36	482 (100)^a^	34	31	Yes	Yes	119 (100)^a^	35	35	Yes	Yes	
2	54	118 (100)^a^	13	7	Yes	Yes	15	1	0	Yes	Partial	
3	48	62	5	1	Yes	No	11	1	1	Yes	Yes	
4	66	52	3	1	Yes	Yes	21	1	0	Yes	No	
5	60	476 (100)^a^	26	22	Yes	Partial	384 (100)^a^	13	13	Yes	Yes	
6	54	149 (100)^a^	8	8	Yes	Partial	59	11	11	Yes	Partial	
7	60	5	1	0	Yes	Partial	11	1	0	Yes	No	
8	60	72	18	10	Yes	Partial	10	0				
9	66	20	4	4	Yes	Yes	9	0				
10	54	31	1	0	No	No	23	0				
11	54	5	0				22	1	0	Yes	Yes	
12	48	32	0				49	0				
13	48	14	0				20	0				
14	54	25	0				16	0				
15	60	12	0				23	0				
16	54	7	0				11	0				
17	60	76	0				37	0				

^a^A maximum of 100 randomly selected pre-detections per patient per band were evaluated; the number of FOs corresponds to the number of true positive pre-detections in these groups of 100

### Review by Experts

**Fig. 2:**
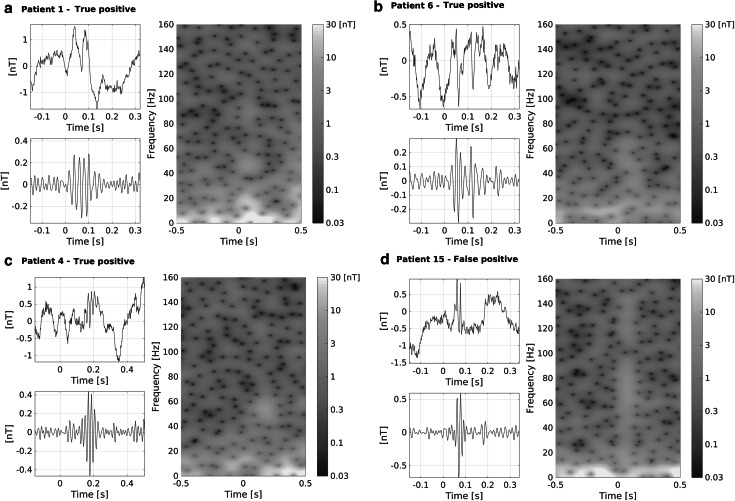
Examples of true positive FOs (**a**–**c**, Patient 1, 6, and 3 respectively) and a false positive pre-detection (**d**, Patient 15) in the high-gamma band. Each *panel* shows the unfiltered MEG signal in a channel where the FO was detected, the same signal pass-band filtered between 40 and 80 Hz, and a time–frequency decomposition of the signal

**Fig. 3:**
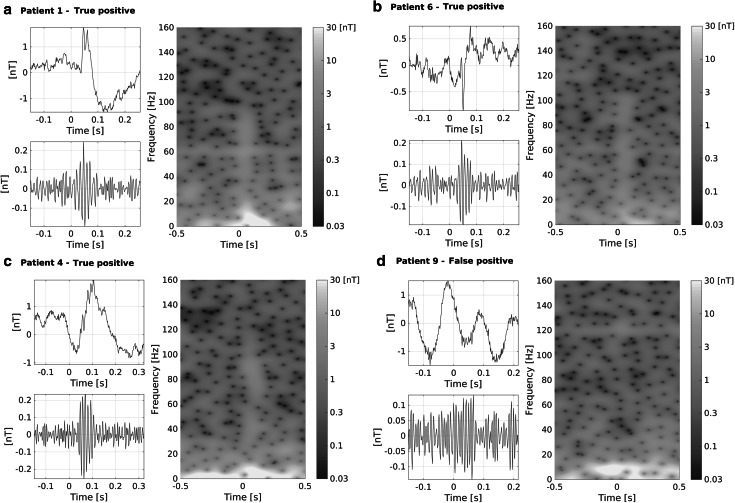
Examples of true positive FOs (**a**–**c**, Patient 1, 6, and 4 respectively) and a false positive pre-detection (**d**, Patient 9) in the ripple band. Each *panel* shows the unfiltered MEG signal in a channel where the FO was detected, the same signal pass-band filtered between 80 and 160 Hz, and a time–frequency decomposition of the signal

The independent review of the pre-detections by the two experts showed a high degree of concordance for both FO bands, with 85 % agreement between reviewers. This evaluation further revealed that the specificity of the automatic detection step was close to 10 % for this group of patients in both bands. This low specificity was expected given the desired high sensitivity of the automatic detection. In some cases, the FOs were clearly visible in the raw unfiltered signal as shown in Figs. 2 and 3, which illustrate raw and filtered MEG signals scored as true and false positive pre-detections in the high-gamma and ripple bands (full panels for each individual patient can be found in Figures S1–S17 of the supplementary material). In five patients we observed that more than half of the FOs occurred at the same time as IEDs (Figs. 2, 3, S1–S17 and in Table 1), with higher rate of FOs-IEDs co-occurrence in the ripple band (93 %) as compared to the high-gamma band (73 %).

After reviewing up to 100 pre-detections per band per patients, the output of the human experts review demonstrated that FOs were confirmed in a total of 11/17 patients: high-gamma band FOs were found in 10/17 patients (range 1–34 per patient), and ripple band FOs were found in 8/17 patients (range 1–35 per patient). Individual values for each patient are given in Table 1. Note that limiting the number of reviewed pre-detections to 100 had no impact in the sensitivity, since in all but one of the cases there were several true positive FOs among the reviewed pre-detections. In the remaining case (patient 17), all the 100 pre-detections were false positives due to extremely noisy background activity, and we concluded that there was no reason to review any additional pre-detections in this case.

In six patients, no FOs were found in any band (patients 12 to 17, Table 1), and four of them were mesial temporal lobe epilepsy patients (patients 13–15, 17). This is not surprising as it is well recognized that the yield of MEG is higher in neocortical epilepsies and more so in extra-temporal foci.

### Rate and Concordance of FOs

The median FO rate in the high-gamma band was 0.02 per minute (range 0–4.55 per minute), and in the ripple band 0 per minute (range 0–1.16 per minute), individual values for each patient are given in Table 2. FO rates for patients in whom the number of pre-detections was larger than 100 were calculated under the assumption that the proportion of true positives for the total initial detections would remain equal to that found for the 100 randomly selected events. The rate in the high-gamma band was larger or equal to that in the ripple band in all but one patient (patient 11), but no statistical significance was found for the difference in median rates between both bands (Wilcoxon rank sum test, *p* = 0.26).

**Table 2:** MEG and EEG highest rate channel

Patient	Epi. region^a^	High-gamma (40–80 Hz) rate (per min) and highest rate channel				Ripple (80–160 Hz) rate (per min) and highest rate channel			
		MEG	Channel	EEG	Channel	MEG	Channel	EEG	Channel
1	RF^b^	4.55	MRT11	8.17	Fp2-F10	1.16	MRF25	0.08	Fp2-F10
2	LPO	0.28	MLT14	2.30	**C3-T3**	0.02	MLT14	0.07	**C3-T3**
3	LT	0.10	MLT14	3.57	P9-O1	0.02	MLF67	4.27	P9-01
4	RTP	0.05	MRT23	1.13	T4-C4	0.02	MRT27	0.33	F8-T4
5	RF^b^	2.06	MRF45	1.20	Fp2-F10	0.83	MRF45	0.53	Fp2-F10
6	LP^c^	0.22	MLP52	0.47	**F7-T3**	0.19	MLP52	0.27	**F7-T3**
7	RT + LT	0.02	MRF65	0.07	T5-O1	0.02	MRP57	0	–
8	LF^b^	0.30	MLC24	0.33	F3-C3	0	–	0.20	Fp1-F3
9	RPO^b^	0.06	MRT27	5.30	P10-O2	0	–	1.97	P10-O2
10	RF	0.02	**MLP51**	0.30	Fp2-F8	0	–	0.03	F8-T4
11	RT	0	–	0.37	Zy1-Zy2	0.02	MRF46	0	–
12	LO + RO	0	–	4.23	T9-P9	0	–	5.67	P9-O1
13	RT	0	–	0.70	F8-T4	0	–	0.13	**Fp2-F10**
14	LT	0	–	0.33	T3-T5	0	–	0.20	**F3-C3**
15	RT + LT	0	–	0.10	**C4-Cz**	0	–	0.10	Zy1-Zy2
16	LFT^b^	0	–	0.10	F9-T9	0	–	0.07	F3-C3
17	RT	0	–	0.20	Zy2-T4	0	–	0.13	**F4-C4**

Channels in bold indicate lack of concordance with epileptogenic region

^a^Epileptogentic region (lobar level) defined from available clinical information (see “Epileptogenic region” section). *R* right, *L* left, *F* frontal, *P* parietal, *O* Occipital, *T* temporal

^b^Patients with confirmed focal cortical dysplasia

^c^Patient with suspected focal cortical dysplasia

There was an excellent lobar level concordance between the topographic maps of the number of detected MEG FOs per channel (as represented in Fig. 1) and the clinical information available for each patient, observed in 10/11 patients with FOs (9/10 patients in the high-gamma band and in 8/8 patients in the ripple band). The only case that was not concordant was patient 10, with right frontal lobe epilepsy and a high-gamma oscillation in the left occipital lobe. Thus, sensitivity was of 53 % and false discovery rate (FDR: number of non-concordant cases divided by number of concordant or non-concordant cases) was 10 % for high-gamma FOs. Lower sensitivity (47 %) and FDR (0 %) were observed in the ripple band. Table 1 includes a summary of spatial concordance at lobar level.

An excellent agreement was found also between channels with highest high-gamma and ripple FO rates. In the seven patients in whom oscillations were found in both bands, the channels with highest number of detected FOs found for both frequency bands overlapped (Fig. 1 and Supplementary Figs. S1–S7). Three patients had high-gamma oscillations but no ripple oscillations, and one patient had one ripple detected but no high-gamma oscillations.

The FO rate in five patients with a confirmed diagnosis focal cortical dysplasia (Patients 1, 5, 8, 9, and 16) was compared to the FO rate in 11 patients without focal cortical dysplasia (Patients 6 was not included in the analysis because of EEG findings consistent with focal cortical dysplasia, but no imaging or pathology results confirming the diagnosis). The median high-gamma band FO rate in focal cortical dysplasia patients was 0.30 per minute, significantly higher than in non-focal cortical dysplasia patients (*p* = 0.02, Wilcoxon rank sum test). In the ripple band the medians of the rates of FOs in both groups were both zero and not statistically different.

### MEG and Scalp EEG FOs Comparison

As this series of patients have undergone scalp EEG FOs evaluation through well-established methods, a comparison with the scalp EEG results serves to further validate our methodology and our MEG findings (Table 2). Scalp EEG FO median rates were significantly higher than MEG rates, both in the high-gamma band (0.47 per minute - Wilcoxon rank sum test, p = 0.0004), and in the ripple band (0.20 per minute—Wilcoxon rank sum test, *p* = 0.002).

The EEG channel with highest FO rate was discordant with the epileptogenic region at lobar level in 3/17 patients for high-gamma band and in 5/16 patients for the ripple band. Of note, in all cases in which the scalp EEG channel with highest rate was not concordant with the epileptogenic region there were still FOs detected in channels that were spatially concordant, albeit with lower rate. In summary, the higher FO rates observed in scalp EEG for the same patients lead to higher sensitivity but lower specificity when compared to MEG, with 82 % sensitivity and 18 % FDR in the high-gamma band, and 65 % sensitivity, 31 % FDR in the ripple band.

### MSI of High-Gamma and Ripple FOs

**Fig. 4:**
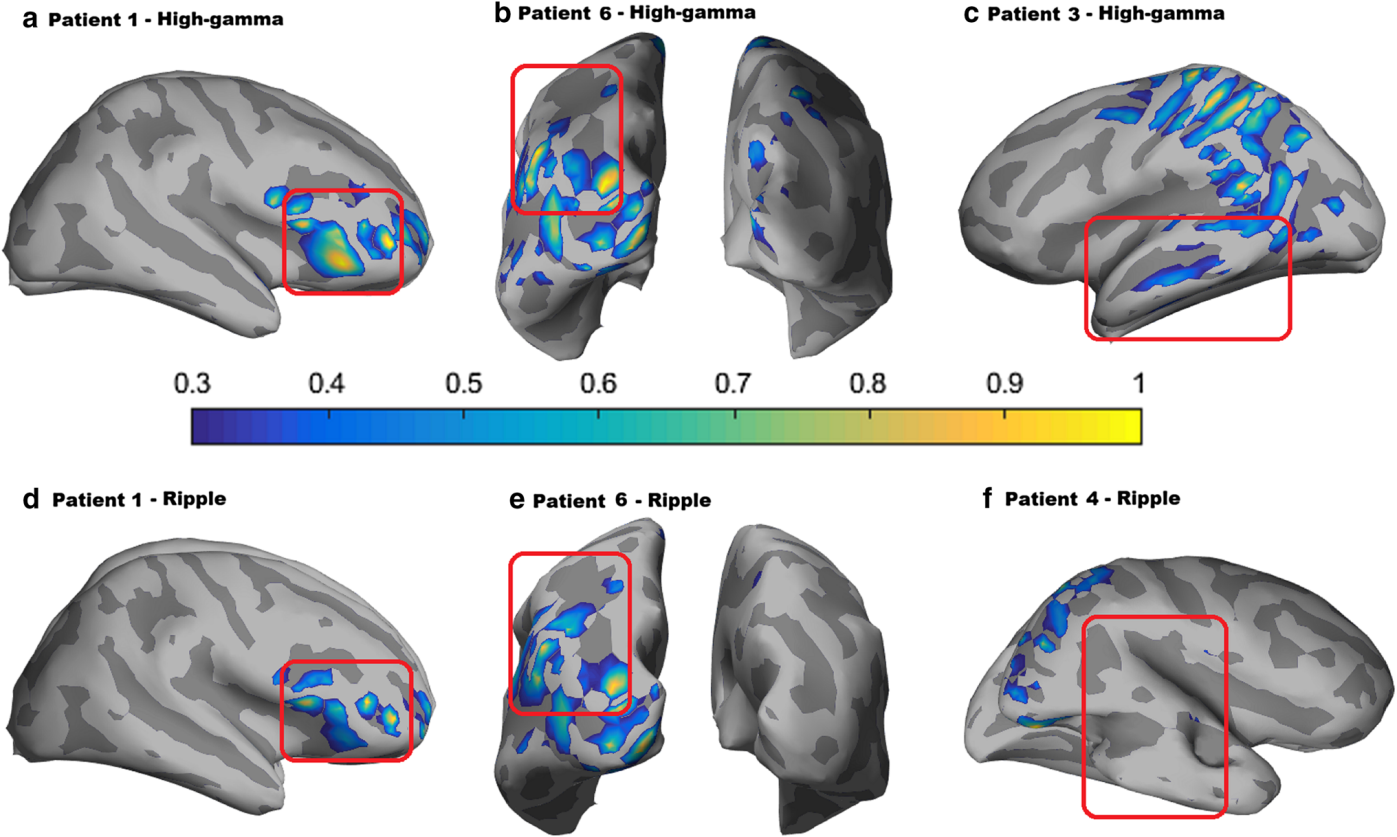
Magnetic source imaging using wMEM of FOs in the high-gamma band (**a**–**c**) and in the ripple band (**d**–**f**). Sources are displayed over inflated cortical surface in a normalized color scale. Epileptogenic region (as determined by two experts based on the available clinical information) for each patient is represented with a red square. For patient 1 (**a** and **d**) FO sources were highly concordant with the epileptogenic region, whereas for patient 6 (**b** and **e**) local maxima and the epileptogenic region partially overlapped. For patient 3 (**c**) and patient 4 (**f**) MSI showed discordance with the epileptogenic region

MSI was performed using wMEM method for all patients with true FOs; i.e. in 10 patients for high-gamma (Fig. 4a–c) and in eight patients for ripple band (Fig. 4d–f). Please refer to Figs. S1–S11 of the supplementary material for individual patients results. Spatial concordance at sub-lobar level between MSI results and available clinical information is included in Table 1. MSI showed sources that were fully concordant and exclusive to the epileptogenic region in 8 maps (4/10 in the high-gamma band and 4/8 in the ripple band).

MSI in the high-gamma band revealed partially concordant sources in 4/10 additional patients (Patients 5–8). In these patients, sources were found within the epileptogenic region but also outside it, as can be seen in Fig. 4c. For patient 8, who has been operated and is not seizure free, the maximum FO source was posterior to the resection, raising the possibility that sources of MEG FOs could have a clinical significance and influence postoperative prognosis. The same partial concordance was observed in 2/8 patients for the ripple band (Patients 5 and 6, Fig. 4e). These two patients with partial concordance in both bands had widespread epileptic activity on scalp EEG and had not undergone iEEG investigation, so the epileptogenic region was determined with less certainty than in the other cases.

Finally, there were 4 MSI maps discordant with the epileptogenic region, in three of them there was only one detected FO so no averaging was possible, in the fourth (high-gamma band in Patient 3) there were three detected FOs. Patient 10 had discordant findings for high-gamma FOs at channel level, and naturally this translated in a discordant map at source level. MSI in the ripple band in patient 7 identified a source at the right inferior frontal gyrus instead of the temporal lobe, but these two locations are very close in Euclidean distance and the inverse solution might have failed to discriminate this anatomical difference due to low SNR. In only 2 of the 18 performed MSI (high-gamma band in patient 3; ripple band in patient 4), the sources were clearly not concordant with the location of the channel in which the FOs were detected (Fig. 4c, d), and could be considered failures of the inverse solution algorithm.

## Discussion

In this series of patients previously described with scalp EEG FOs (Andrade-Valença et al. 2011; Melani et al. 2013), we demonstrated that it was possible to record FOs non-invasively with MEG and to analyze them using semi-automatic detection followed by 3D source localization. Identification of FOs in the high-gamma and ripple bands was possible despite acquisitions of limited duration that preclude obtaining ideal sleep recordings as done with scalp EEG and iEEG studies using overnight recordings. Patients were not able to reach slow wave sleep and many did not sleep at all during the 1-h recording session, leading to an increased possibility of artifacts and to a reduced rate of HFOs (Bagshaw et al. 2009; Staba et al. 2004). The possibility to validate our MEG and MSI findings with the scalp EEG data in these patients was an important step in the challenging non-invasive evaluation of FOs using MEG signals.

Because MEG signals are recorded with hundreds of sensors, it is not feasible in practice to detect FOs visually by human experts. The combination of an automatic detector with high sensitivity followed by validation by human raters is a practical approach, appropriate for the high number of channels and low FO rates encountered in MEG. We also showed for the first time that it was possible to perform MSI and to identify cortical generators of MEG ripples. Due to the high number of sensors in MEG as compared to scalp EEG, determining non-invasively the sources of these potential biomarkers of the epileptic focus through MSI is of clinical relevance. Although source localization results of single FOs can be affected by noise given the low SNR of the signals, the performance improves by averaging the results in source space as we have done in this study, even if only a few events are available.

### Detection

As for scalp and iEEG FOs, some of the detected MEG FOs were visible in the unfiltered signals, with a large proportion occurring simultaneously with IEDs, indicating that the events were not effect of filtering and are likely of epileptic origin.

The sensitivity for this heterogeneous group of patients was of 53 % in the high-gamma band and 47 % in the ripple band, but with extremely low FO rates. As can be seen in the topographic maps (Fig. 1) we found a very good agreement between the MEG channels exhibiting the highest FO rate and the epileptogenic region. At channel level we identified only one likely case of physiologic oscillation that fulfilled our definition of FO (patient 10), in the high-gamma band and recorded from the occipital region, where high rates of physiologic HFOs of high amplitude and long duration can be recorded intracranially (Matsumoto et al. 2013; Wang et al. 2013).

The rate of FOs in the high-gamma band in our study was quite lower than the 2.2 per min average rate reported by Jeong et al. (2013), but the mentioned MEG study included only focal cortical dysplasia patients and in our cohort some of the focal cortical dysplasia patients have rates in the same range.

In the ripple band, we found FOs in eight patients, but in five of them only one event was identified in the whole recording time. This extremely low rate is consistent with the findings of van Klink et al. (2015), who report FOs in MEG channels of 3 out of 12 patients during recordings of 15 min duration. The higher sensitivity in our study would then be explained simply by the longer recording time.

### Comparison Between High-Gamma and Ripple Band FOs

In this study, there was an excellent agreement between channels with highest high-gamma and ripple FO rates. The main difference we found between the analyzed FOs was the lower rate in the ripple band compared to the high-gamma band. The same observation was previously reported for scalp EEG FOs (von Ellenrieder et al. 2014), and the proposed explanation remains valid for MEG: higher frequency oscillations have lower amplitude, either because the generators are smaller or because the synchronization of neural populations is more difficult at higher frequencies. As the noise at these frequencies is dominated by the white noise of the amplifier, i.e. its amplitude is relatively constant in these high frequency bands, this results in lower SNR and reduced detectability of FOs at higher frequencies.

Even though FOs in the high-gamma band could also have less specificity for localizing epileptic activity due to the possibility of physiologic oscillations, as mentioned above we only found one likely physiologic FO in the high-gamma band (patient 10). Jeong et al. (2013) report a much higher rate of physiologic gamma oscillations (around 40 % of the detected events), but the difference could be attributed to the fact that we restricted our study to the highest FO rate channel, which is likely to be related to epileptic activity.

### Comparison Between MEG and Scalp EEG FOs

The comparison between MEG and scalp EEG in the same patients showed higher FO rates for scalp EEG, leading to higher sensitivity but also lower specificity, indicating that higher rates are not necessarily more desirable in non-invasive evaluation of FOs. It is important to note that the scalp EEG recordings corresponded to non-REM sleep stages (N2 and N3) from recordings in video-EEG monitoring in our unit. MEG recordings did not have this restriction. Many patients slept during the MEG recording, but only during part of the study and in most cases not reaching deep sleep. This could partly explain the difference in FO rates, since it has been established that HFO rates increase during these deeper sleep stages (Staba et al. 2004; Bagshaw et al. 2009). In a recent study the mean HFO rates in the neocortex increased during deep sleep compared to wakefulness in values ranging from 21 % for the frontal lobe to 69 % for the temporal lobe (Dümplemann et al. 2015). In addition, during wakefulness muscle artifact is more likely to occur and they might overlap with FOs further reducing the detectable rates, even if less so in MEG than in scalp EEG. Also, part of the FOs visible in scalp EEG could arise from generators with an almost radial orientation, precluding them from being identifiable in MEG due to the low sensitivity to these type of sources. Finally, the number of detected FOs will depend on their SNR. If the noise level is comparable to the amplitude of the oscillations the detection will not be possible. Because the instrumentation noise is different in EEG and MEG, this could be another explanation for the difference in FOs rate. In this regard, the high spatial sampling of MEG compared to low density clinical EEG represents an advantage since with a higher number of sensors there is a higher chance to be recording closer to the maximum field, increasing the chance of having a channel where FOs can be recorded with higher SNR.

### MSI of High-Gamma and Ripple FOs

MSI of MEG FOs is a challenging problem given the low SNR of these events. The usual approach in low SNR signals is to average the events before solving the inverse problem. This approach can be used when the timing of the events is known, e.g. in stimulation studies (Papadelis et al. 2009) or spikes with similar morphology in epilepsy. However, in the case of pathologic FOs the timing is random, and even a small error in the estimated timing can lead to a significant cancellation of these high frequency signals. Thus, an optimized method to achieve reasonable performance in source localization of single events is needed. Then, the averaging can be done at source space level. The wMEM method applied in this study has two particularities that make it especially suited for this problem. First, the discrete wavelet decomposition allows for denoising before solving the inverse problem, and a very parsimonious decomposition is expected for high-frequency oscillatory phenomena such as the FOs (Lina et al. 2014). Selecting only the wavelet coefficient with highest absolute amplitude for the source localization takes advantage of these facts, allowing to focus on the discharge of interest in the frequency band of interest, while limiting noise contamination for these low SNR signals. The ability of wMEM to provide accurate source reconstruction from the wavelet coefficient of one time–frequency box exhibiting maximum energy has been evaluated using realistic simulations (Lina et al. 2014), and is applied on clinical data for the first time in this study.

The other important property of the MEM framework is that the model is based on a parcellation of the cortex together with hidden state variable allowing “to switch off” inactive parcels while being able to reconstruct accurately the spatial profile of the sources within the active parcels (Amblard et al. 2004, Lapalme et al. 2006). In the present study, we expect MEM to “switch off” many parcels, resulting in a null activation of many regions of the cortex, especially when the SNR is low. This shutting off instead of risking incorrect localization is convenient for averaging single event localization maps, minimizing the contribution of brain regions that are not involved in the generation of the FOs.

Overall, good results were obtained with MSI using the described methodology, even when the number of events per patient was very low. In only 2 out of 18 cases MSI was discordant with the location of the channels where the FOs were detected. This can be explained by the simultaneous presence of noise in the frequency bands of interest and could perhaps be improved by removing noisy MEG channels. This approach was not explored in the present study.

In two other cases MSI was concordant with the location of the channels with FOs but outside the epileptogenic region. In general, the low SNR of the FOs can explain the unsuccessful source localizations. In several patients there were multiple FOs sources, sometimes extending beyond the epileptogenic region. These many local maxima could be due to slightly different source localization results for each event before averaging, as can be expected for low SNR signals. But it could also reflect the involvement of different generators, since FOs with the same scalp topology can be generated by different intracranial sources (Zelmann et al. 2014). It is possible that MEG and MSI are able to unravel these different generators given that the blurring effect of the skull is not as important as for EEG and thus these multiple clustered sources might be in fact the ground truth, as suggested by a simultaneous intracranial investigation of HFOs with cortical grids and scalp EEG (Zelmann et al. 2014).

To our knowledge this is the first study performing MSI of spontaneous and likely pathological MEG FOs on the ripple band. One previous study investigated sources of gamma band using the sLORETA method, and found 60 % concordance with the epileptogenic region in patients with an underlying focal cortical dysplasia (Jeong et al. 2013). However, there is not enough information to decide if the incorrect localization of the remaining cases was because the detected events were of physiologic origin as the authors claim, or due to shortcomings of the source localization approach.

### Potential Avenues for Future Studies

The straightforward way to improve the sensitivity in MEG FOs detection would be by extending the recording time to increase the number of detected events. Longer recording times would possibly also increase the proportion of sleep in the recordings, with the consequent increase in the FO rates. This approach is highly constrained by individual patients’ tolerance.

Another way to improve the sensitivity could be to relax the specification of four clear oscillations for the definition of FOs, adopted in our study based in the extensive results available in the intracranial EEG literature in which this definition is adopted. It is entirely possible that a less strict definition of FOs could be more useful in the context of MEG studies, but it will decrease specificity. This should be further investigated since many pre-detections could reflect IEDs with high-frequency spectral content, which could nevertheless also be useful for identifying the epileptogenic region (Guggisberg et al. 2008). Alternatively, the sensitivity can also be increased with the use of virtual channels (van Klink et al. 2015), although ideally a way to define the virtual channels without relying on IEDs would be important, to avoid bias in the results.

Source localization could also be improved by fusion of the simultaneous scalp EEG and MEG recordings, which was not pursued in this study. The complementary information of these modalities has been shown to improve the reliability of the source localization in general (Muravchik and Nehorai 2001) and in particular for the MEM method (Chowdhury et al. 2015).

The main objective of this study was to introduce this framework for the detection and source localization of FOs in MEG. The selection of a heterogeneous group of focal epilepsy patients (Supplementary Table) further validates the robustness of the proposed methodology. A more in-depth prospective evaluation of the clinical value of MEG FO analysis in focal epilepsy could further corroborate its role in as a biomarker for the epileptogenic region and as a predictive factor for outcomes.

## Conclusion

We have demonstrated that the identification of FOs in MEG is feasible from a practical point of view. Compared to scalp EEG FOs, the rates in MEG are lower but with the advantage of a higher specificity. We have also shown that it is possible to localize the source of these oscillatory events with high spatial resolution. This opens interesting potential applications for the non-invasive study of FOs not only in a large population of epilepsy patients, but also extending the study of physiologic fast oscillations to healthy controls.

### Electronic supplementary material

Supplementary material 1 (PDF 8249 kb)

Supplementary material 2 (PDF 201 kb)

## Appendix

### Resampling

The change in sampling frequency was done in two steps: (i) upsampling by a factor of 2 or 4 followed by filtering and downsampling by a factor of 3 to an intermediate sampling frequency of 1600 Hz and (ii) upsampling by a factor of 2 followed by a downsampling by a factor of 5 to a final sampling frequency of 640 Hz. The antialiasing/smoothing filters were finite-length impulse response filters of order 80.

### Automatic Detector

The detector relies first in a wideband filter (band-pass finite impulse response filters, equiripple, order 200, transition bands of 10 Hz width, pass band from 40 to 160 Hz, weighting factor 10 on the low frequency stop band), and 10 narrow bands filters in the 40–160 Hz range (40–46–53–61–70–80–93–107–123–142–160 Hz), five in the high-gamma band and five in the ripple band (band-pass finite impulse response filters, equiripple, order 500, transition bands of 5 Hz width, weighting factor 10 on the low frequency stop band).
