# Supplementary material for: Detection and Magnetic Source Imaging of Fast Oscillations (40–160 Hz) Recorded with Magnetoencephalography in Focal Epilepsy Patients
Source: Brain Topogr. 2016 Jan 30;29:218–31. doi: 10.1007/s10548-016-0471-9 (PMC4754324; doi:10.1007/s10548-016-0471-9)
Supplement: Supplementary file 1 — Supplementary material 1 (PDF 8249 kb) [file 10548_2016_471_MOESM1_ESM.pdf]

Supplementary material

Figures S1 to S17: Results for Patients 1 to 17 respectively.

A – G: Results for the high-gamma band. A) Example of several channels at the time of a pre-detection in the high-gamma band, pass-band filtered between 1 and 70 Hz. The amplitude scale is given by the red bar in the bottom left corner, and corresponds to 1 nT. B) Unfiltered signal in a channel where a pre-detection occurred, with expanded time axes. C) Idem B filtered between 40 and 80 Hz. D) Time-frequency decomposition of the signal shown in B. E) Number of pre-detections in each MEG channel simultaneous with the pre-detections in the channel with highest rate. The red circle indicates the channel corresponding to panels B, C, and D. The red and black circles correspond to the channels shown in panel A. The front of the head is at the top.

If any of the pre-detections in the high-gamma band were true positive FOs:

F) Number of FOs in each MEG channel simultaneous with the FOs in the channel with highest rate. G) Source localization result. Normalized average of the energy in the single event source localizations. The red rectangle indicates the epileptic region as evaluated by two experts based on the available clinical information.

H – N: Results for the ripple band. H) Example of several channels at the time of a pre-detection in the ripple band, pass-band filtered between 1 and 70 Hz. The amplitude scale is given by the red bar in the bottom left corner, and corresponds to 1 nT. I) Unfiltered signal in a channel where a pre-detection occurred, with expanded time axes. J) Idem I filtered between 80 and 160 Hz. K) Time-frequency decomposition of the signal shown in I. L) Number of pre-detections in each MEG channel simultaneous with the pre-detections in the channel with highest rate. The red circle indicates the channel corresponding to panels I, J, and K. The red and black circles correspond to the channels shown in panel H. The front of the head is at the top.

If any of the pre-detections in the ripple band were true positive FOs:

M) Number of FOs in each MEG channel simultaneous with the FOs in the channel with highest rate. N) Source localization result. Normalized average of the energy in the single event source localizations. The red rectangle indicates the epileptic region as evaluated by two experts based on the available clinical information.

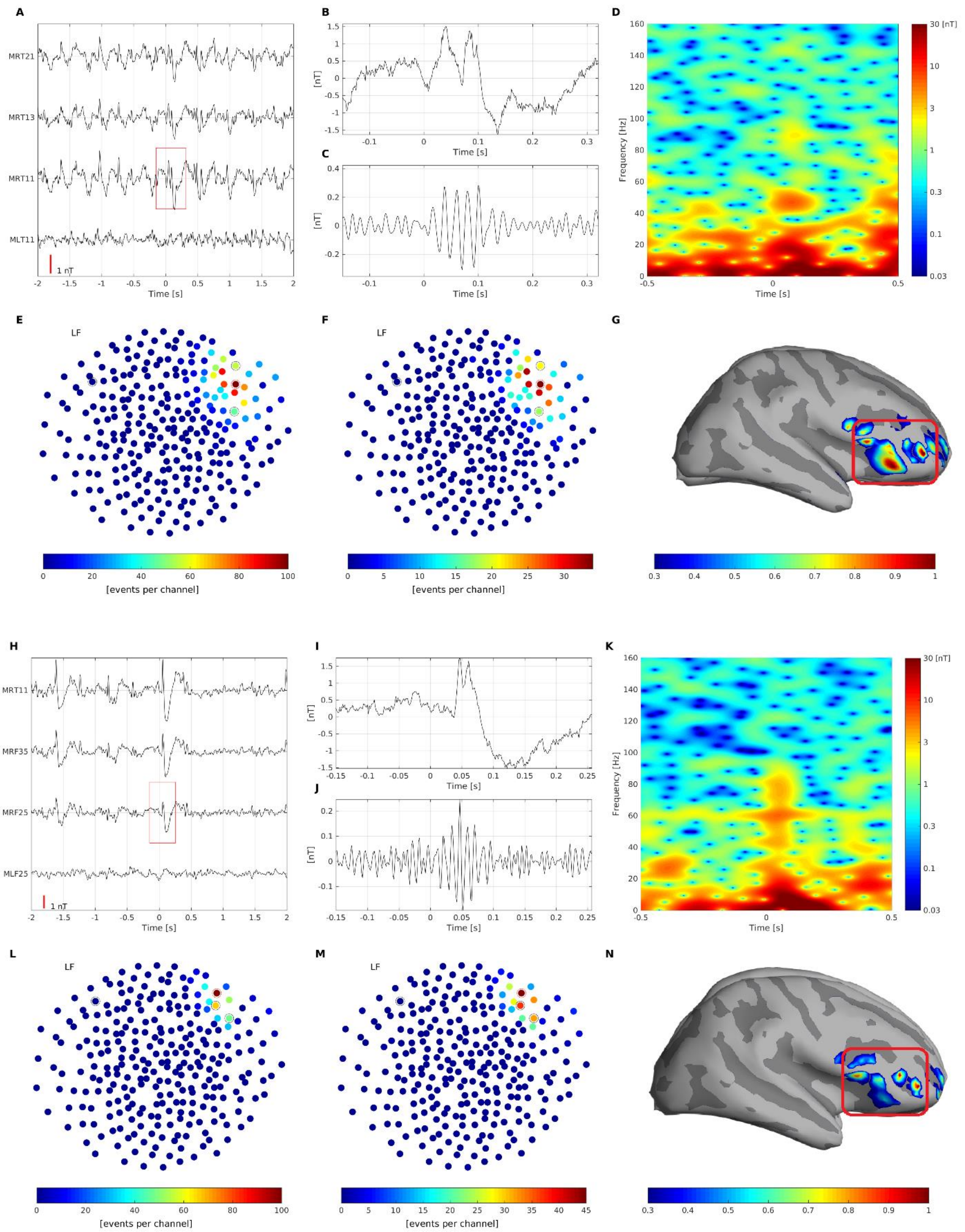

Figure S1: Patient 1, right frontal epilepsy.

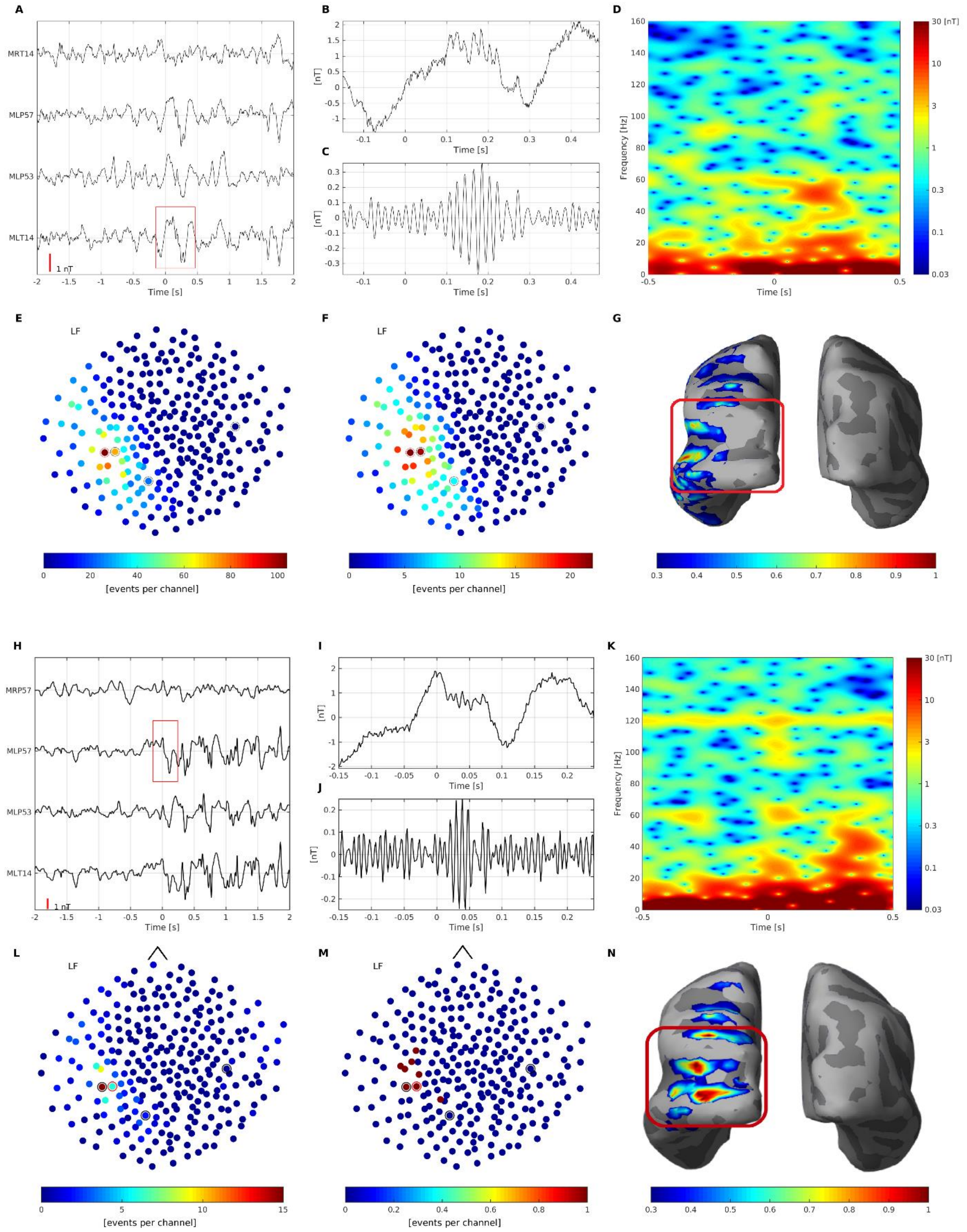

Figure S2: Patient 2, left parieto-occipital epilepsy.

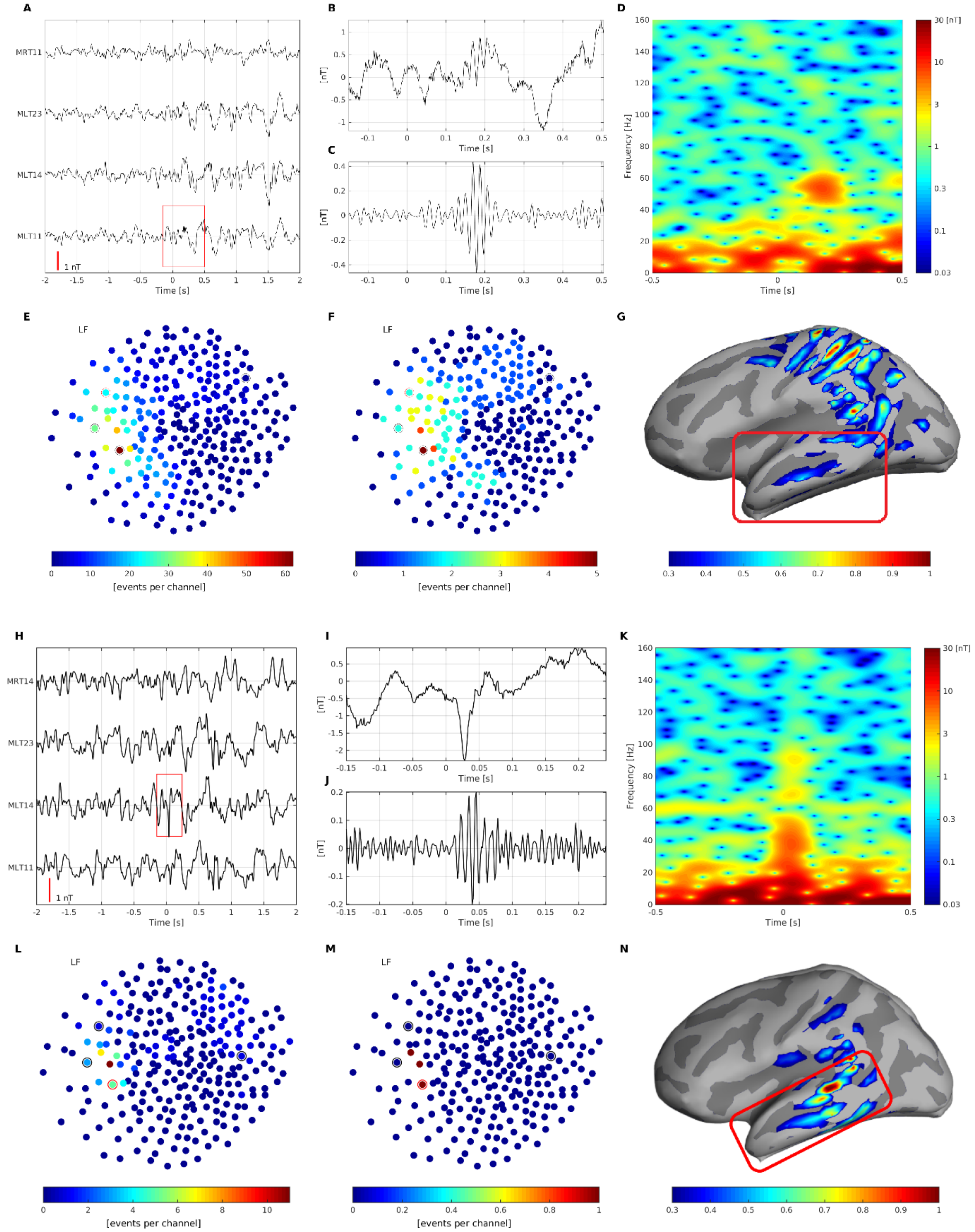

Figure S3: Patient 3, left temporo-parietal epilepsy.

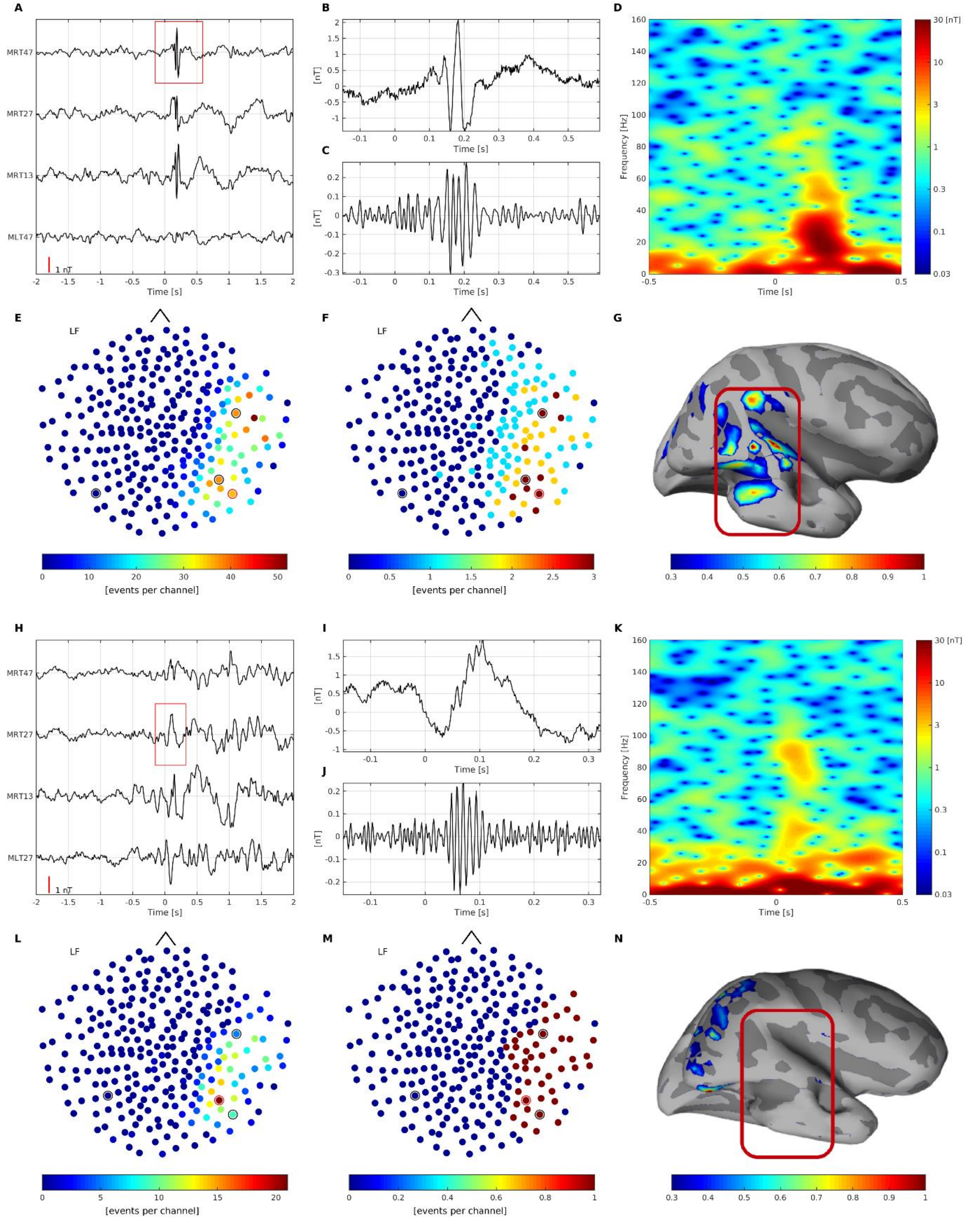

Figure S4: Patient 4, right temporo-parietal epilepsy.

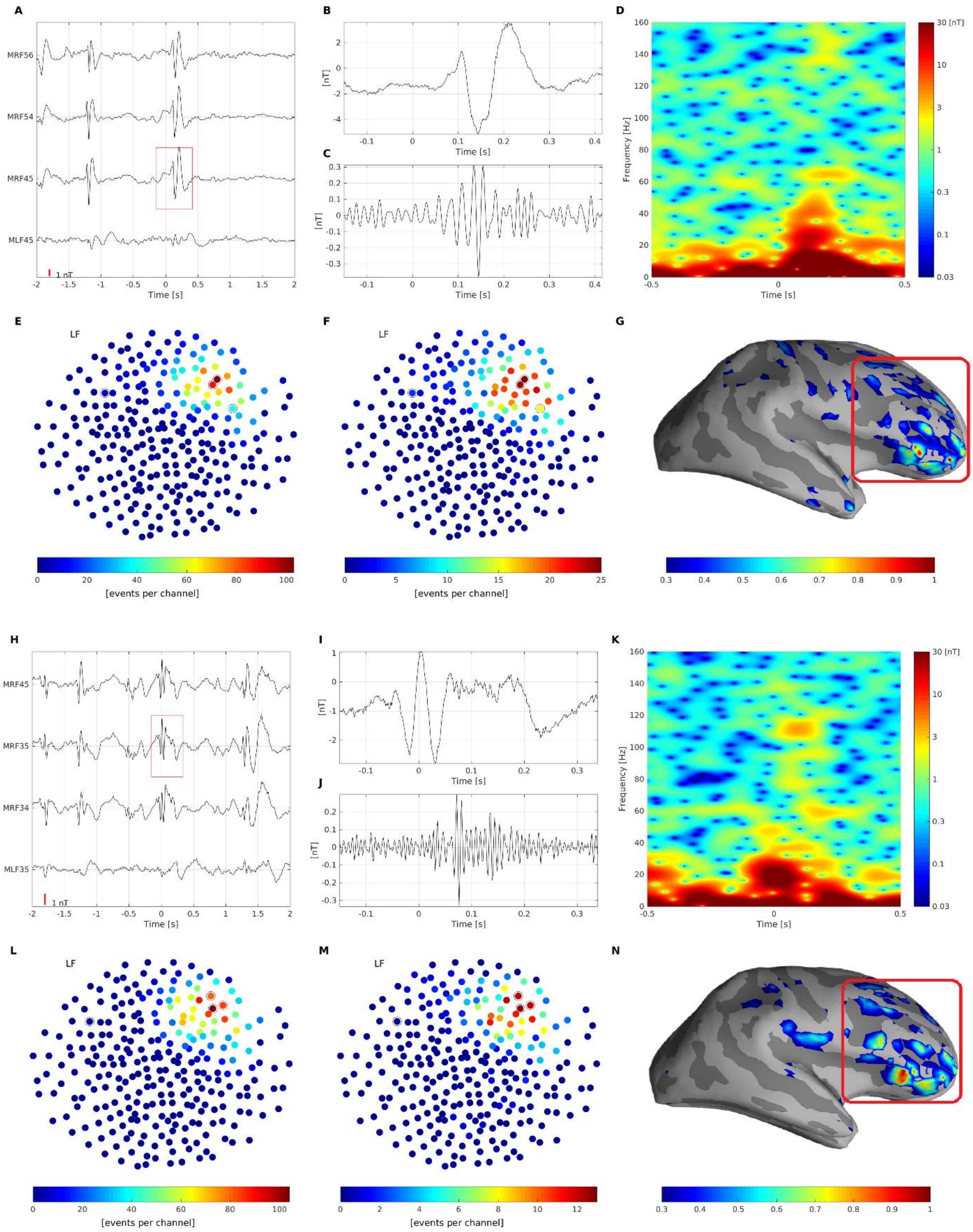

Figure S5: Patient 5, right frontal epilepsy.

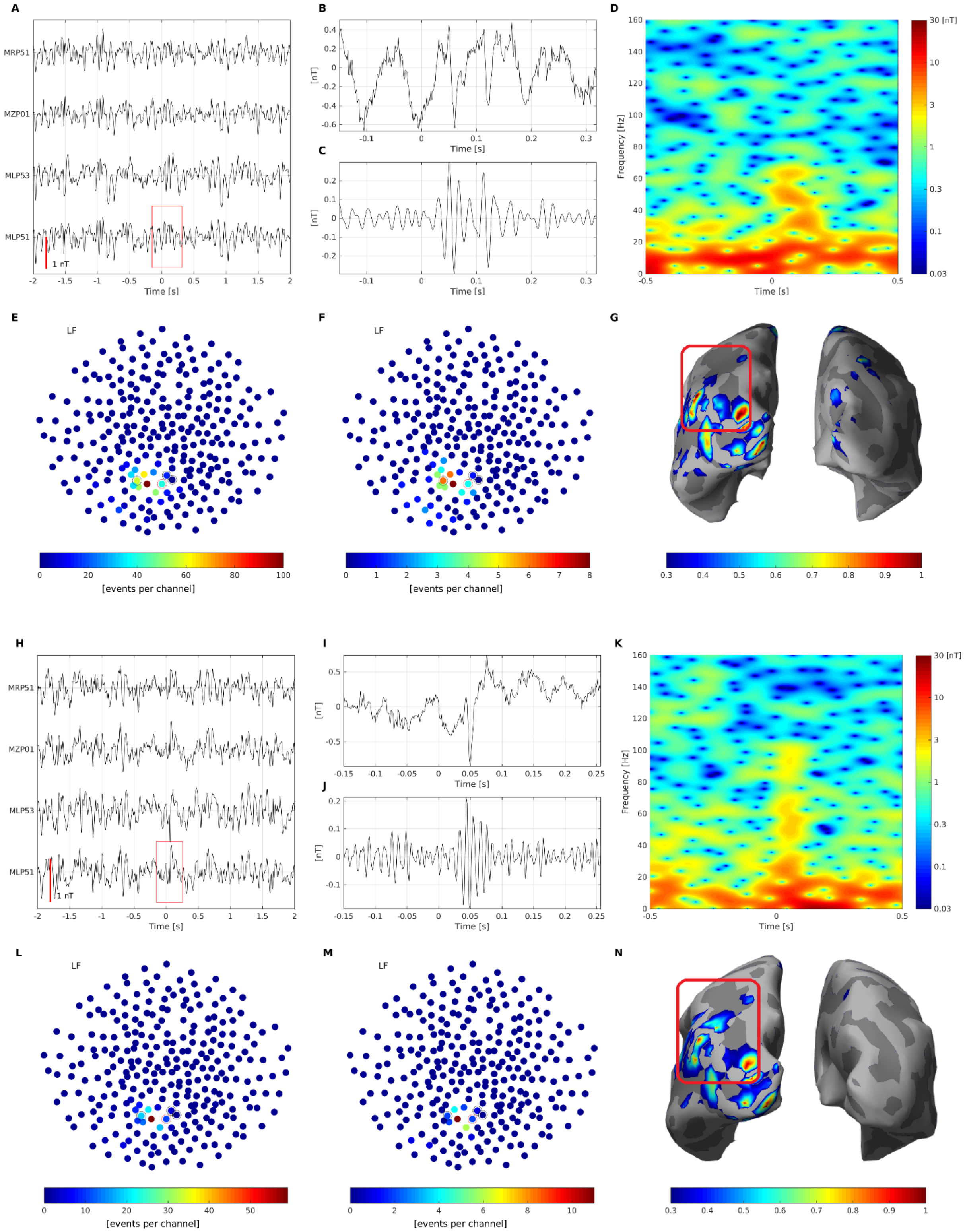

Figure S6: Patient 6, left parieto-occipital epilepsy.

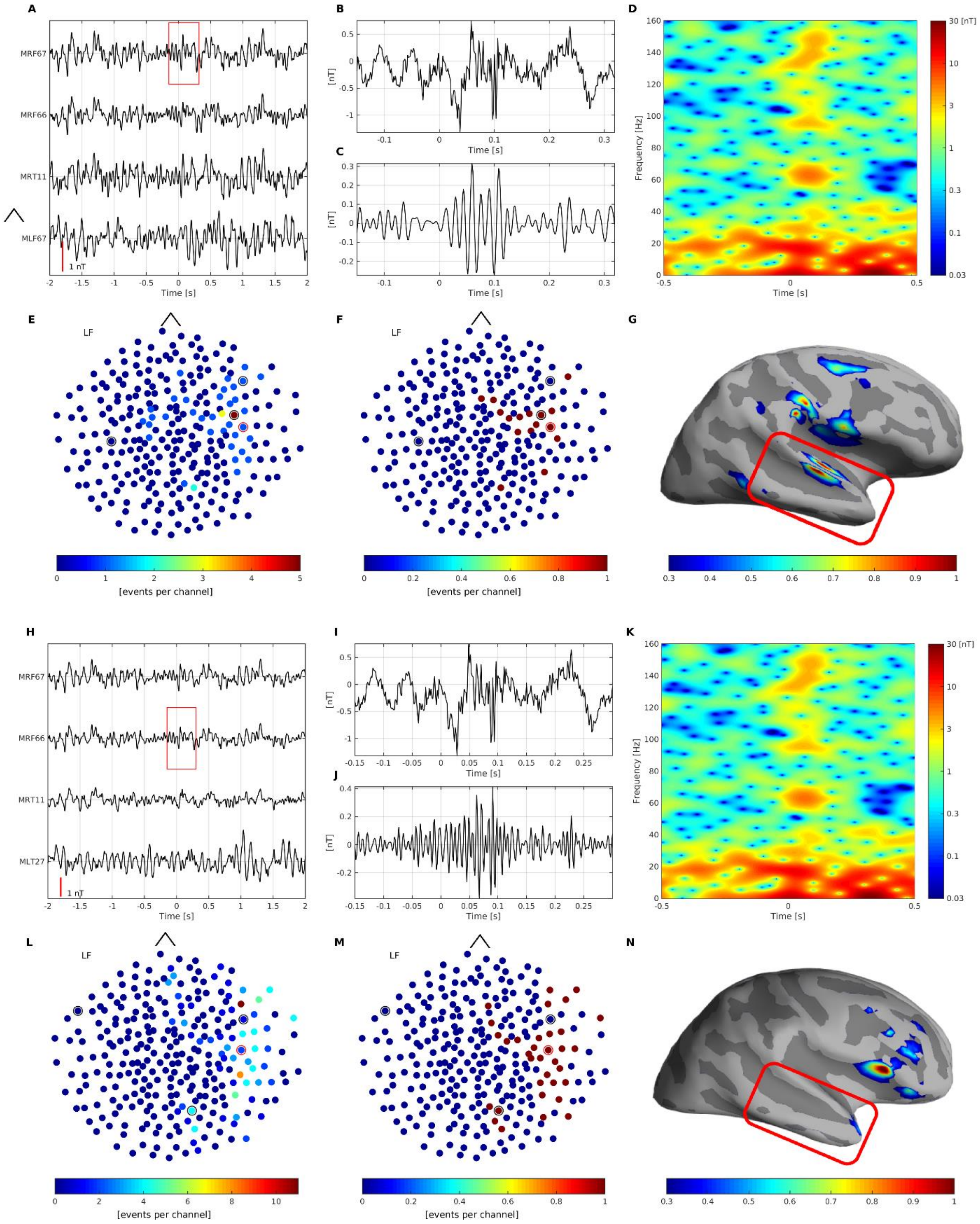

Figure S7: Patient 7, right bi-temporal epilepsy.

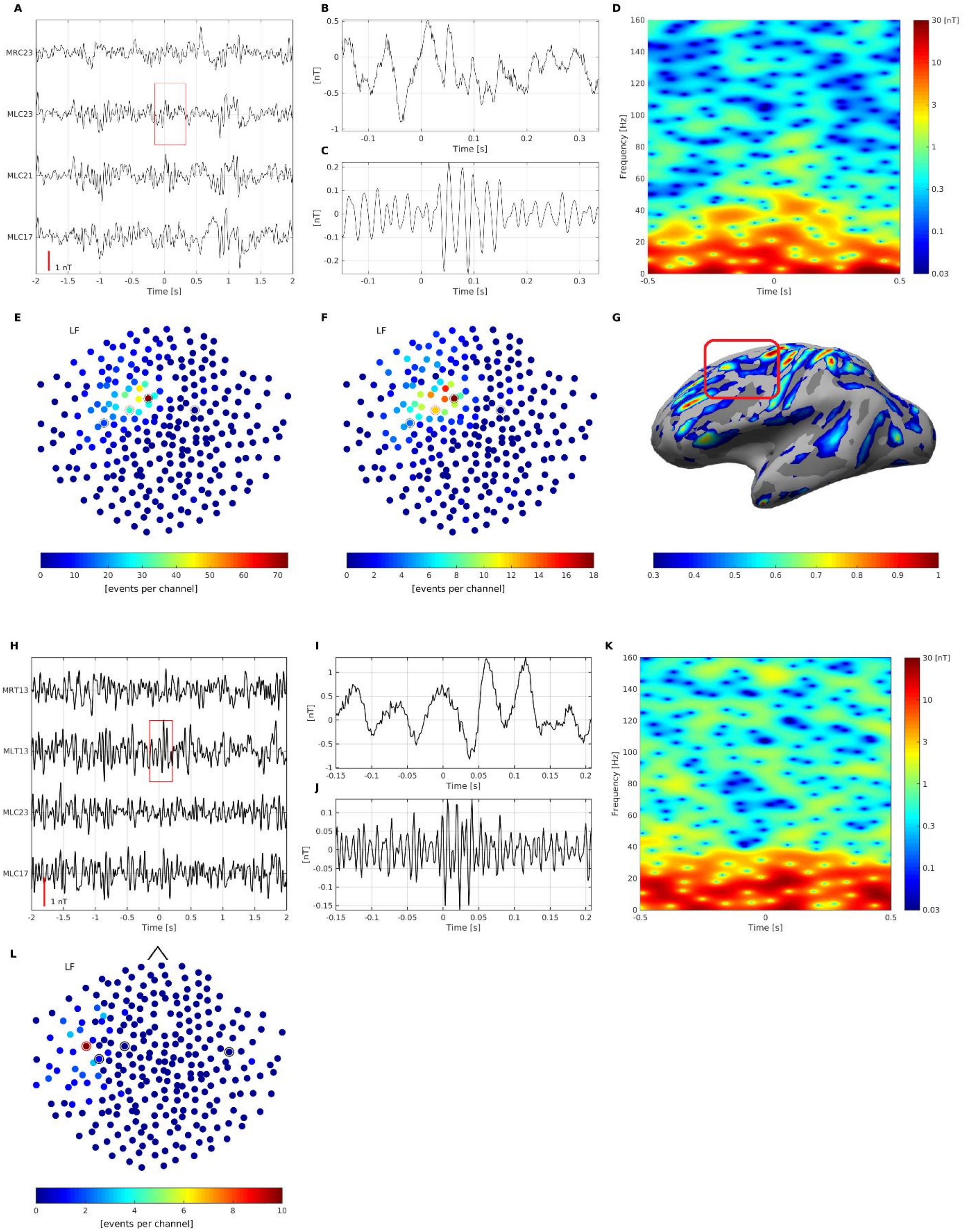

Figure S8: Patient 8, right frontal epilepsy.

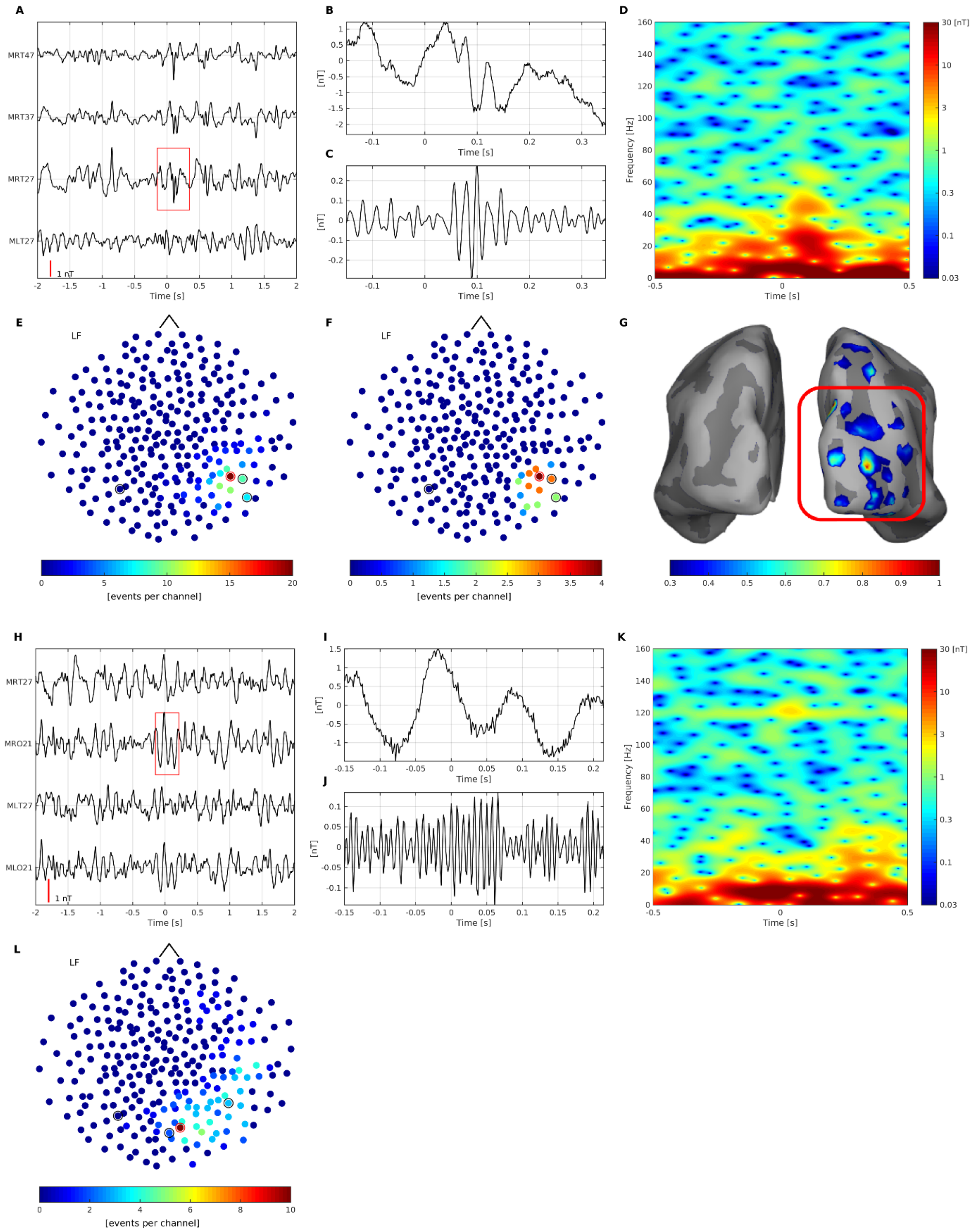

Figure S9: Patient 9, right parieto-occipital epilepsy.

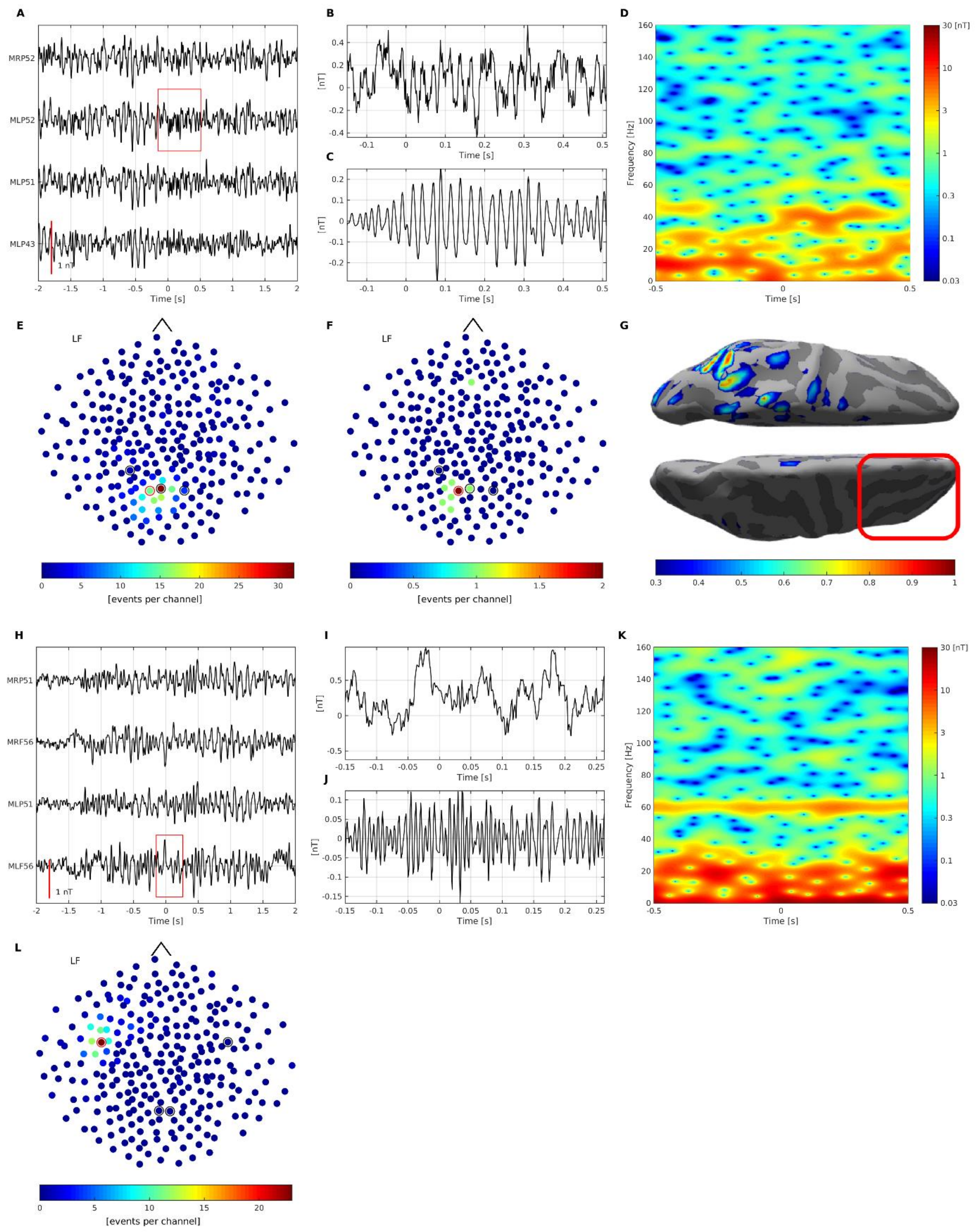

Figure S10: Patient 10, right frontal epilepsy.

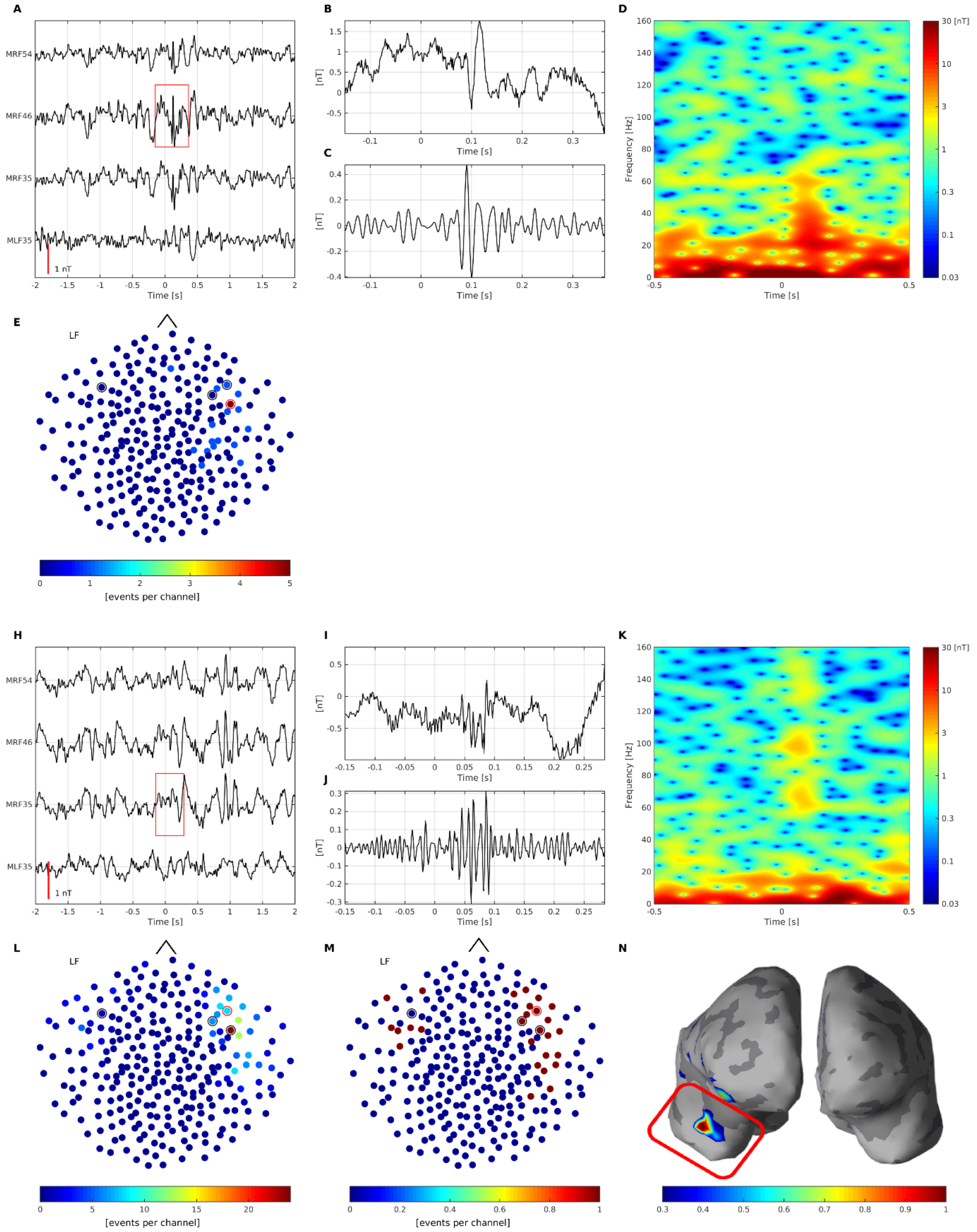

Figure S11: Patient 11, right temporal epilepsy.

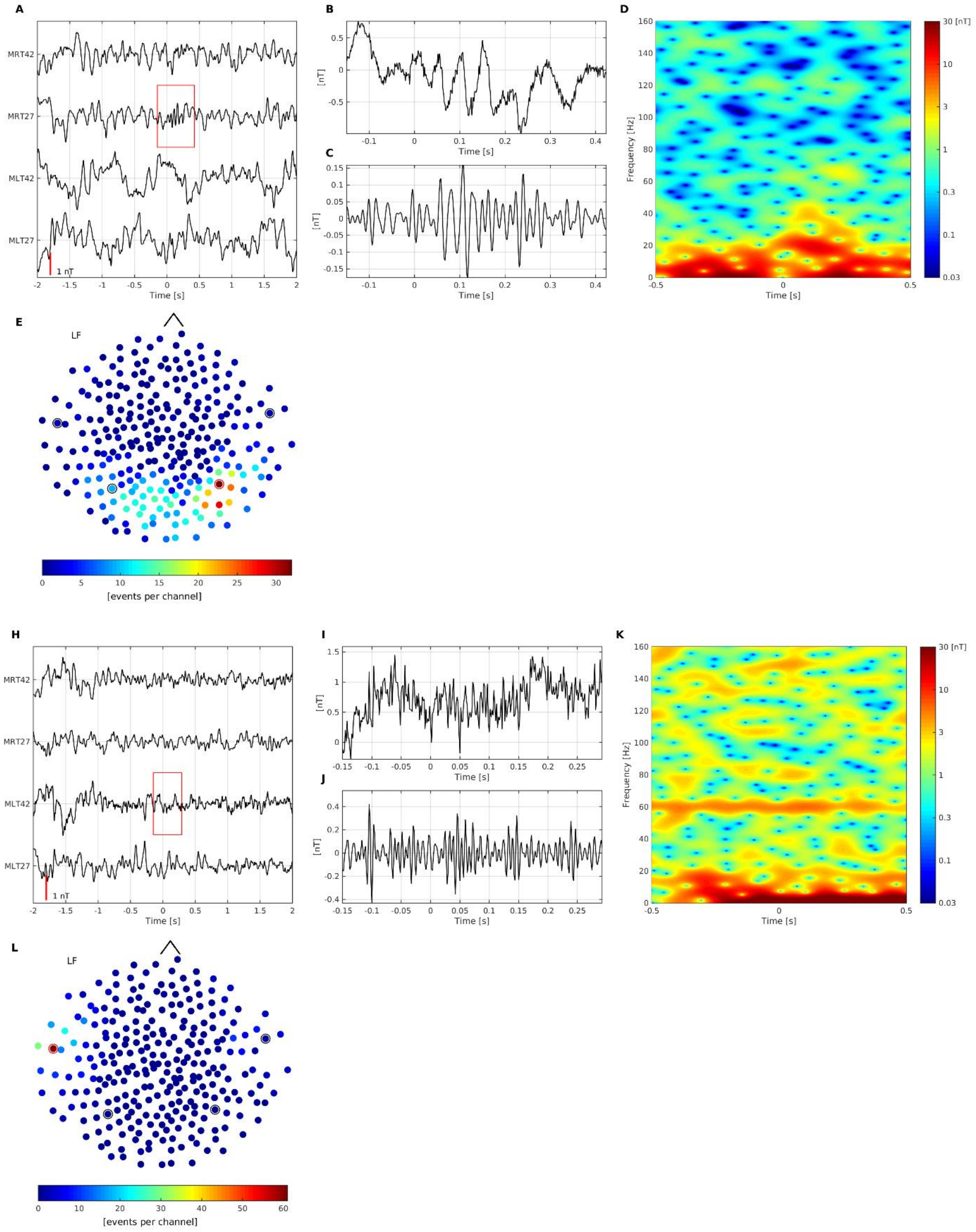

Figure S12: Patient 12, occipital epilepsy.

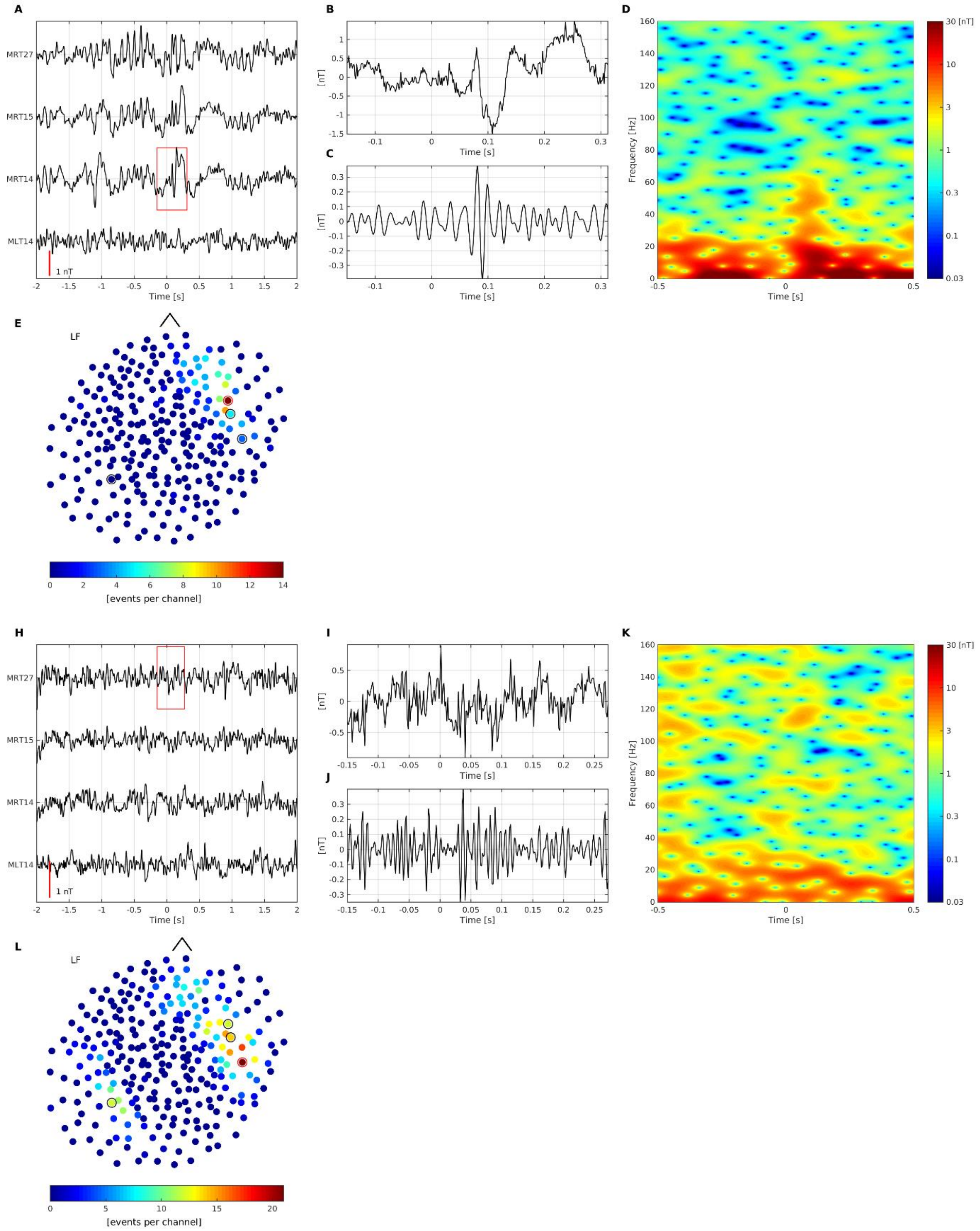

Figure S13: Patient 13, right temporal epilepsy.

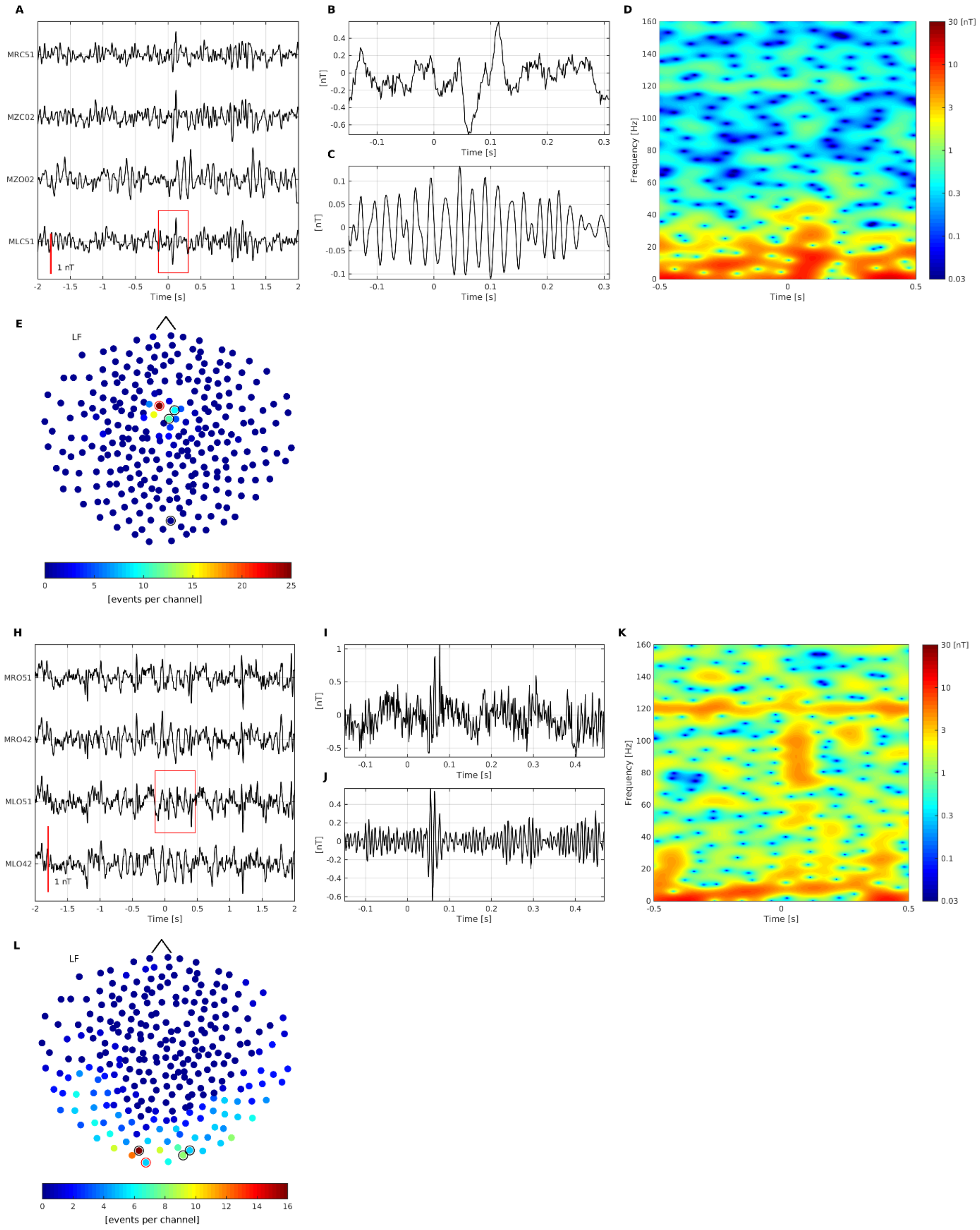

Figure S14: Patient 14, left temporal epilepsy.

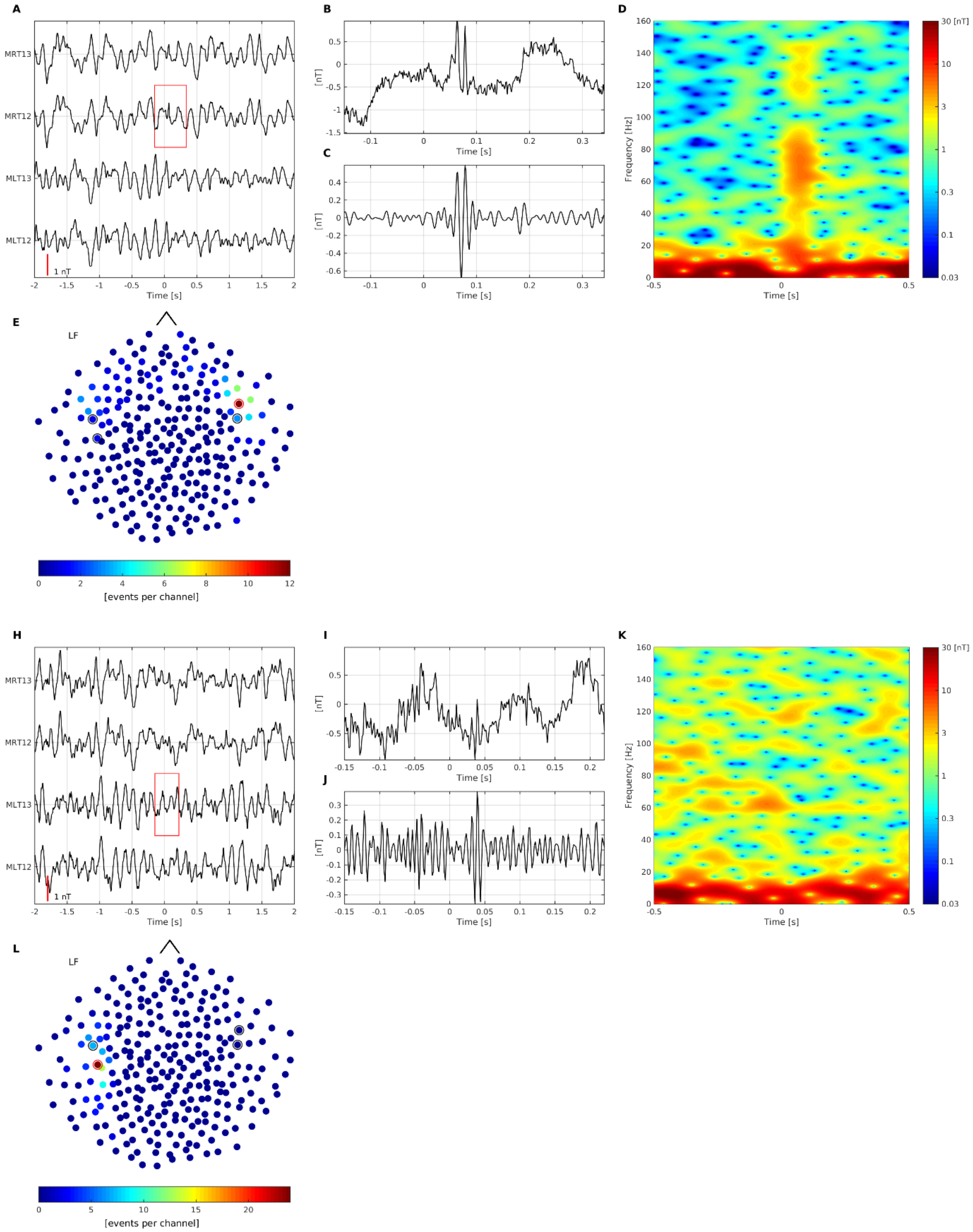

Figure S15: Patient 15, bi-temporal epilepsy.

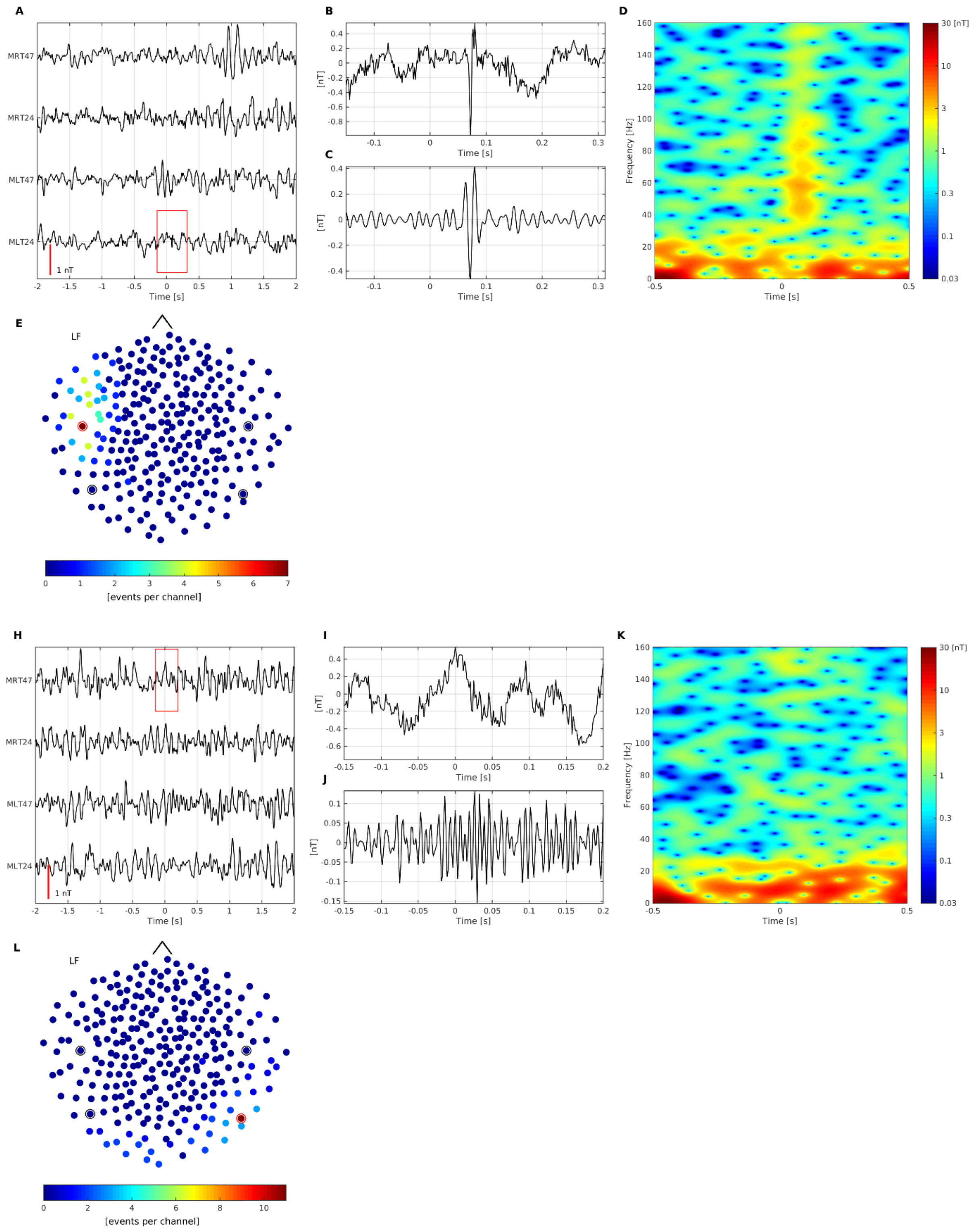

Figure S16: Patient 16, left fronto-temporal epilepsy.

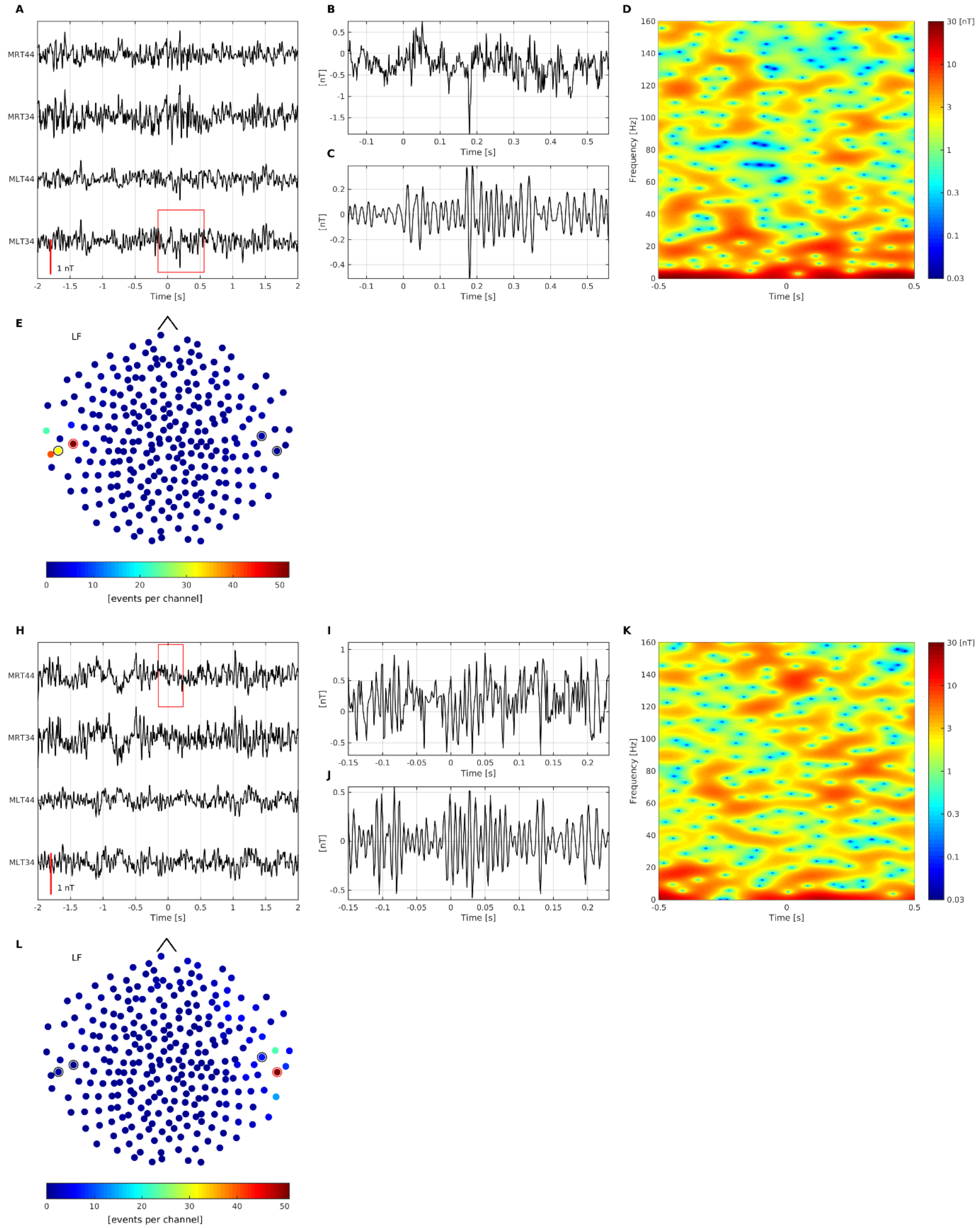

Figure S17: Patient 17, right temporal epilepsy.
