# Supplementary material for: Detection and Magnetic Source Imaging of Fast Oscillations (40–160 Hz) Recorded with Magnetoencephalography in Focal Epilepsy Patients
Source: Brain Topogr. 2016 Jan 30;29:218–31. doi: 10.1007/s10548-016-0471-9 (PMC4754324; doi:10.1007/s10548-016-0471-9)
Supplement: Supplementary file 2 — Supplementary material 2 (PDF 201 kb) [file 10548_2016_471_MOESM2_ESM.pdf]

Supplementary Table I: Clinical Information.

Patient	Surgery	Outcome	iEEG Interictal	iEEG Ictal	Scalp Interictal	Scalp Ictal	MRI
**1**	Right orbitofrontal resection	Engel I	Right orbitofrontal	Right orbitofrotal	Right fronto-temporal and frontal	Right fronto-polar and frontal	Right orbitofrontal focal cortical dysplasia
**2**	Palliative mini-invasive surgery	Engel III after first procedure, only one month FU after second procedure	3 independent regions: 1) post. insula involving temporal neocortex, 2) left occipital pole and supracalcarine region, 3) left hippocampus	Supracalcarine electrodes and post. insula	Widespread left temporo-parietal maximum at T7-P7	Left temporo-parietal	Multifocal nodular heterotopia involving the left posterior temporal, parietal and occipital regions
**3**	n.a.	not operated, not seizure-free	n.a.	n.a.	Left temporal, Involving anterior and posterior temporal	Left posterior temporal	Non lesional
**4**	Right temporal resection with further extension of the resection	Engel III	n.a.	n.a.	Left temporo-parietal	Right centro-temporal-parietal	Right temporo-parietal heterotopia
**5**	n.a.	Not operated, not seizure-free	Interictal activity involving both frontal lobes, more often over the right frontal regions	Ill defined, Very widespread right frontal generator, ictal activity at times starting from the left frontal	Frontal atypical spike and wave discharges more prominent on the right	Bilateral frontal maximum at right	Right frontal focal cortical dysplasia

				regions			
**6**	n.a.	Not operated, seizure free with medication	n.a.	n.a.	Left posterior quadrant, maximum P3	left posterior parietal maximum P3	Left posterior quadrant atrophy
**7**	n.a.	not operated, not seizure-free	Bilateral mesial temporal	Bilateral mesial temporal	Bilateral temporal	Bilateral temporal	Left hippocampal atrophy
**8**	Left frontal resection	Engel III	Bilateral frontal, max in the lesion and perilesional	Bilateral frontal, max in the lesion and perilesional	Bilateral fronto-central maximum left	Left mid frontal	Left frontal focal cortical dysplasia
**9**	Right posterior quadrant resection	Engel II	Widespread discharges over the right posterior, parietal and occipital regions	Ill defined, over the right posterior parietal and occipital regions	widespread over the right hemisphere	Right posterior quadrant	Right posterior quadrant focal cortical dysplasia
**10**	Right frontal resection	Engel II	Right fronto-temporal	Right orbitofrontal	Anterior head regions	Bilateral frontal maximum right	Right frontal focal cortical dysplasia
**11**	Right selective amygdalo-hippocampectomy	No updated follow-up information available	n.a.	n.a.	Right temporal	Right temporal	Right hippocampal atrophy
**12**	n.a.	not seizure-free	n.a.	n.a.	Bilateral posterior quadrant maximum left	Bilateral maximum left	Left posterior quadrant focal cortical dysplasia
**13**	Right temporal resection	Engel I	n.a.	n.a.	Right temporal	n.a.	Right hippocampal atrophy

**14**	Left amygdalo hyppocampectomy followed by extension of resection	No follow-up info after second surgery in 2012	Left mesial temporal	Left hippocampus	Left temporal	Left fronto-temporal	Non lesional
**15**	Right temporal resection sparing the hippocampus	Engel II	Bilateral mesial temporal	Bilateral temporal maximum right.	Right frontotemporal and Left temporal	Right temporal	Bilateral hippocampal atrophy
**16**	Left orbitofrontal resection	Engel I	Left insular, orbitofrontal, temporal and opercular	Left Orbitofrontal	Left fronto-temporal.	Left temporal	Left orbitofrontal focal cortical dysplasia
**17**	Right selective amygadlo-hippocampectomy	Engel I	n.a.	n.a.	Right temporal.	Right temporal	Right hippocampal atrophy and gliosis. Left middle cranial fossa meningoencephalocele.
